# Analysis of in vivo single cell behavior by high throughput, human-in-the-loop segmentation of three-dimensional images

**DOI:** 10.1186/s12859-015-0814-7

**Published:** 2015-11-25

**Authors:** Michael Chiang, Sam Hallman, Amanda Cinquin, Nabora Reyes de Mochel, Adrian Paz, Shimako Kawauchi, Anne L. Calof, Ken W. Cho, Charless C. Fowlkes, Olivier Cinquin

**Affiliations:** 1 Department of Developmental & Cell Biology, University of California at Irvine, Irvine, USA; 2 Center for Complex Biological Systems, University of California at Irvine, Irvine, USA; 3 Department of Anatomy & Neurobiology, University of California at Irvine, Irvine, USA; 4 Department of Computer Science, University of California at Irvine, Irvine, USA

**Keywords:** Spatial cytometry, 3D image segmentation, Stem cells, Cell cycle, *C. elegans* germ line, Mouse pre-implantation embryo, Olfactory placode, Olfactory epithelium

## Abstract

**Background:**

Analysis of single cells in their native environment is a powerful method to address key questions in developmental systems biology. Confocal microscopy imaging of intact tissues, followed by automatic image segmentation, provides a means to conduct cytometric studies while at the same time preserving crucial information about the spatial organization of the tissue and morphological features of the cells. This technique is rapidly evolving but is still not in widespread use among research groups that do not specialize in technique development, perhaps in part for lack of tools that automate repetitive tasks while allowing experts to make the best use of their time in injecting their domain-specific knowledge.

**Results:**

Here we focus on a well-established stem cell model system, the *C. elegans* gonad, as well as on two other model systems widely used to study cell fate specification and morphogenesis: the pre-implantation mouse embryo and the developing mouse olfactory epithelium. We report a pipeline that integrates machine-learning-based cell detection, fast human-in-the-loop curation of these detections, and running of active contours seeded from detections to segment cells. The procedure can be bootstrapped by a small number of manual detections, and outperforms alternative pieces of software we benchmarked on *C. elegans* gonad datasets. Using cell segmentations to quantify fluorescence contents, we report previously-uncharacterized cell behaviors in the model systems we used. We further show how cell morphological features can be used to identify cell cycle phase; this provides a basis for future tools that will streamline cell cycle experiments by minimizing the need for exogenous cell cycle phase labels.

**Conclusions:**

High-throughput 3D segmentation makes it possible to extract rich information from images that are routinely acquired by biologists, and provides insights — in particular with respect to the cell cycle — that would be difficult to derive otherwise.

**Electronic supplementary material:**

The online version of this article (doi:10.1186/s12859-015-0814-7) contains supplementary material, which is available to authorized users.

## Background

Understanding the mechanisms by which cells make proliferation and differentiation decisions is a question of key interest to systems, developmental, and stem cell biologists. Individual cells display rich cycling and differentiation behaviors that are often not deterministic — as illustrated by stochastic transitions between different progenitor states [1–3] — and that are obscured in population averages. Furthermore, cell proliferation and differentiation are controlled to a large degree by extracellular cues that often can be only very partially and crudely reproduced in vitro. To better understand the mechanisms underlying cell proliferation and differentiation, new tools are thus required to quantify the behavior of single cells in their native tissue environments.

Most techniques currently used to quantify properties of individual cells — such as flow cytometry — rely on tissues being dissociated prior to analysis, which destroys the spatial and morphological information present in the sample. These sources of information are preserved by imaging of undissociated tissues or organs; such imaging can be performed readily with current technologies (e.g. confocal microscopy), but it does not immediately lead to cell-by-cell information without extensive analysis to segment individual cells in the resulting three-dimensional (3D) images.

Here we report the overall methodology that we have followed to study the spatial distribution of cell cycle or cell differentiation properties in three different tissues: the *C. elegans* germ line, the mouse pre-implantation embryo, and the mouse olfactory epithelium. While there is an ever growing set of biological image segmentation software solutions that tackle this problem, we found that the parameters of these systems were often difficult to tune and that most did not offer the capability to manually curate intermediate results during processing. To achieve accurate in vivo cytometry, we thus chose to develop our own software, built on proven, robust algorithms for image analysis, to maintain maximal flexibility in the integration of automated processing and manual labeling effort.

A number of general image segmentation tools exist that are specifically targeted at biological applications, including both open source [4–18] and commercial software (e.g. Imaris, Bitplane or Volocity, PerkinElmer). For more extensive surveys, see e.g. [18–20]. Despite rapid development (see e.g. cell tracking benchmark competition [21]), the problem of automatically producing high-quality 3D segmentations of cells in general images remains unsolved, due to the wide variation in appearance across different tissue and cell types, labeling procedures and imaging methods. Rather than tuning existing pipelines or developing custom segmentation algorithms that might improve performance on images of particular cell types, we decided to design a pipeline that maximizes the utility of the most accurate but most expensive resource in image segmentation: time spent by users providing *ab initio* detections or correcting computer-derived detections. This pipeline aims to provide automation of repetitive tasks for which there is no need for user input (such as applying image transformations, e.g. blurring with pre-determined parameters or segmenting out the region around a putative cell location), and to allow the user to focus on the tasks that provide the highest added value.

We designed our pipeline Parismi (Pipeline for Automated oR Interactive SegMentation of Images) around a simple, two-step idea. Cells are first detected, and these detections are then used to seed a segmentation algorithm. Detection can be performed manually (using a 3D browsing interface similar to e.g. VANO [22]) or by a machine learning algorithm trained from a set of manual annotations used to bootstrap the procedure. We chose a machine learning procedure, somewhat similar to e.g. Ilastik [9] and distinct from ad-hoc processing of the fluorescence signal (e.g. [23–26]), to facilitate reuse across sample types that vary in nuclear morphology and imaging conditions; we use machine learning in a different way than Ilastik in that we do not train the algorithm to separate foreground and background pixels, but rather to identify cell centers. The output of the machine learning algorithm can be reviewed and corrected by the same interface — possible operations are addition and deletion of detections, as well as detection re-centering. As the set of segmented cells that have received manual curation expands, the machine learning algorithm can be re-trained from these segmentations, providing for iterative improvements in the quality of the automatic detection step. This approach is loosely similar in concept to “semi-supervised learning” [27] and “active learning” [9, 28], although our current implementation is not fully interactive in that sense.

As the second step of our segmentation procedure, we use “active contours” (implemented following [29]), which are closed surfaces that are initialized from the detected center point and grow smoothly outwards in three dimensions until they encounter the putative cell boundary (suggested by membrane staining) or until they collide with surfaces corresponding to neighboring cells. The surface evolution is governed by both membrane staining (also referred to as the guide image) and by the curvature of the surface itself; penalizing high local curvatures helps the surface maintain a roughly spherical shape, which provides robustness e.g. to noise in the guide image. In the case of stains that are not limited to the periphery of the structure being segmented, such as DAPI or Hoechst stains for nucleus segmentation, pre-processing of the image can be used to produce a guide image that outlines boundaries, so that active contours can still be applied.

**Fig. 1:**
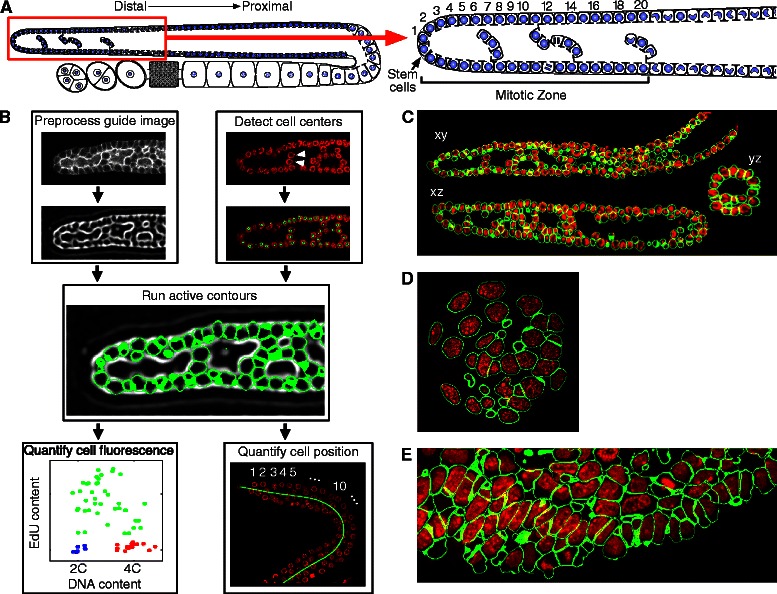
Overview of Parismi image analysis pipeline. **a** Diagram of the *C. elegans* gonadal arm (left) with an enlarged view of the MZ (right). Cell rows are numbered with row one at the distal most end where the stem cells reside. **b** Application of Parismi to images of *C. elegans* gonadal arms. The membrane image is preprocessed using a principal curvature approach to produce a guide image. Cell centers are identified manually or using an SVM classifier, and act as seeds for active contours that are run against the guide image. Position and fluorescence contents are quantified using segmentation masks derived from the active contours. Arrowheads give an example of cells whose DNA staining gap in the nucleus center is larger than the gap between each cell. **c**-**e** Example 3D segmentations of a *C. elegans* gonadal arm (**c**), a pre-implantation mouse embryo (**d**), and mouse olfactory epithelium (**e**); red: DNA channel; green: segmentation mask boundary

The use of active contours has a long history in cell segmentation and has proven to be a robust approach for identifying cellular and nuclear volumes in three dimensions [29–35]. Other approaches to segmentation such as geodesic distance transform [16], gradient flow smoothing [36], and watershed transform have also been used successfully to perform 3D volumetric segmentation of cell nuclei in specific sample preparations (e.g. [37–39]); however, these techniques often require post-processing ([40]; although see [41]) to correct segmentation errors. In particular, segmenting densely labeled whole cells (rather than nuclei) requires high-quality membrane staining to achieve sufficient local contrast [34]. Our choice of seeded segmentation and active contours avoids difficulties that commonly arise in purely segmentation-based approaches based on local filtering and enhancement followed by connected components analysis. In our *C. elegans* gonadal arm data, the spatial distribution of DNA towards the periphery of the nucleus results in gaps (see e.g. Fig. 1 and Additional file 1: Figure S1) in the DNA stain channel that can be larger than the separations between neighboring nuclei and that hence cannot be easily resolved by local smoothing. Active contours do well in that context.

Our choice of seeded segmentation also makes it straightforward to inject domain-specific knowledge of expert users in an efficient way. Tools already exist that enable manual editing of segmentations. ITK-snap [42] targets annotation of a few large volumes (e.g. in medical imagery) and is hence not well adapted to our datasets. Fiji’s “segmentation editor” [14] enables fully-manual editing of segmentation products that, even with the help of interpolation, is particularly taxing in terms of time and effort (see below). VANO [22] is highly interesting in that it allows importing of pre-existing segmentations and fast editing using deletion or merging of segments, and adding adjustable spheres to segments (VANO also allows annotations of segmented cells, as discussed in more detail below). Our approach differs from that of VANO in that we offer a batch processing feature that allows a user to quickly curate multiple datasets without manual setup of each image stack, and more importantly in that human input takes place mid-way in the segmentation process, at a point where it is most efficiently provided. Although in the future it may be desirable to integrate VANO functionality to edit the final segmentation product, we have found that, at least for our purposes, active contour output does not need editing as long as each cell is correctly detected (see Results and Discussion section for performance quantification). It is more efficient to simply edit the locations of cell detections than it is to provide a manual segmentation from scratch, or to start from a completed segmentation and correct all of the erroneous pixels that can result from a single mis-detection (put simply, a stitch in time saves nine). In addition, and importantly, manual curation results can be used to further train the detection algorithm and increase its performance, to the point where manual curation may no longer be required (as we show below).

In summary, Parismi is composed of four broad components: (1) an interface to manually annotate cells in 3D images, and to curate automatic detections if desired; (2) an automatic cell detector that can be trained from manual annotations; (3) an active contour implementation that produces cell segmentations; and (4) a number of plugins for analysis of segmented cells to e.g. quantify their position in the organ and their fluorescence contents (Fig. 1). Overall our procedure is similar to previous reports in that it relies on machine learning for segmentation of biological images (see e.g. Ilastik [9] or Trainable Weka [14]) but distinct in three respects. First, it provides for full automation of repetitive steps, which has allowed us to segment hundreds of thousands of cells. Second, it relies on active contours instead of thresholding, watershed or pixel classification as foreground or background; we have found that active contours provide more robust segmentations when cells are tightly packed and/or not perfectly separated by a clean boundary signal. Third, it allows the user to easily curate results if desired, by editing the set of cell detections and re-running the active contours segmentation — rather than relying on perfect detections that must be achieved by time-consuming fine-tuning of training or segmentation parameters that apply in the same way throughout the image.

To illustrate the utility of this methodology in accelerating biological image analysis, we focus chiefly on the *C. elegans* gonad, an organ that is especially amenable to experimental manipulation and imaging and provides a powerful model system for understanding stem cell niches [43, 44]. The *C. elegans* reproductive system is organized as “gonadal arms”, which form a tube with a long “distal-proximal” axis; stem cells are located at the distal end in a mitotic zone (MZ; Fig. 1a). Based on the analysis of tens of thousands of MZ cells, we derive the following results: (1) germline stem cells spend a larger fraction of cell cycle time in the G2 phase than other cycling cells (see also [45]), (2) germ cells appear to arrest immediately in their current phase of the cell cycle upon starvation, and (3) cell cycle phase can be identified using morphological features. We also derive results on the preimplantation mouse embryo (Fig. 1d), addressing the relationship between two transcription factors associated with different cell fates; and on the olfactory placode (Fig. 1e), where we show that cells in different regions of this rapidly-growing and -involuting epithelial structure have different cell-cycle properties. Overall, our results illustrate the powerful information that can be extracted from 3D images of tissue to analyze cell-cycle and cell-differentiation properties of individual cells.

## Results and Discussion

### Benchmarking cell detection accuracy, segmentation accuracy, and fluorescence quantification accuracy

To develop and validate our cell segmentation technique, we used a dataset consisting of confocal stacks of intact *C. elegans* gonadal arms in which nuclei had been labeled with DNA stain (Fig. 1b-c; see “Datasets” and “*C. elegans* gonadal arm image acquisition” in Methods). We generated a “ground truth” dataset of ~4,500 cell center locations by manually clicking on the centers in xy, xz, or yz planes using Parismi’s graphical user interface (see “Overall software organization” in Methods). A subset of these annotated images were used to generate examples for training the system to automatically detect cell centers while the remainder was used to benchmark the accuracy of various intermediate steps of our processing pipeline.

First, we evaluated the accuracy of automated cell detection (see “Cell detection” in Methods for implementation details). A cell detection was considered a true positive if it was within 1.5 μm (approximately one cell radius) of a manually-annotated cell center and there were no other detections closer to the manually annotated center (cell size and cell spacing in the tissue were such that no pair of ground truth annotations is closer than 1.5 μm). Otherwise, a cell detection was considered a false positive. A manually-annotated center with no cell detections within a 1.5 μm radius was considered a false negative. Our automatic cell detector contains a tunable threshold τ; for high thresholds, the detector returns only a few detections and naturally achieves high precision (few false positives) at the expense of low recall (many false negatives). To summarize detector performance in a manner independent of τ, we computed precision and recall at all thresholds and report the average precision (AP), the area under the precision-recall curve (see Methods). We trained the detector on one experimental dataset composed of twenty MZ image stacks (from wildtype individuals at larval stage L4 + 1 day; total of 7,131 cells), then applied our classifier across twelve independent experimental samples composed of worms of different genotypes, stages of development, and feeding or mating treatments (Additional file 2: Table S1). In these twelve samples, the detector achieved an average AP of 98.7 % ± 2 % in the MZ (Additional file 2: Table S1). Visual inspection showed that most errors were associated with condensation of DNA during M-phase. Although our datasets consist mainly of gonadal arms in which only the MZ was imaged, we also measured detector performance in seven whole gonadal arm images. Detection performance decreased when evaluated on the whole gonadal arm as opposed to its MZ subset, with average AP = 90.6 % ± 9 % (Additional file 2: Table S2). This is likely due to wider range of nuclear morphologies in the proximal germ line that results from meiotic progression (Additional file 1: Figure S1). The range of nuclear morphologies in the gonadal arms is substantially more varied than typically seen in other tissues or organs. Thus, although limitations remain in the application of models trained on one kind of cells to other kinds of cells (as highlighted by an outlier gonadal arm with a lower accuracy of 70 %, Additional file 2: Table S2), overall the relatively high AP over the whole gonadal arm suggests that the automated nuclear detection does generalize.

To quantify the amount of training data needed for good detector performance, we also trained the detector on varying sized subsets of the twenty training images. The detector was trained on each subset and then evaluated on the test dataset. On average, a detector trained with only a single MZ image achieved an average AP of 95.7 % ± 4 % (Additional files 3 and 4: Figure S2 and S3A). Average AP quickly reached a plateau, reaching 99.5 % ± 2 % at eight stacks (Additional file 3: Figure S2); we note that, not surprisingly, this average AP is slightly higher than the 98.7 % value reported above when testing across various experimental conditions. Altogether, these results demonstrate that our automatic cell detector is remarkably accurate in the MZ, while being robust to different experimental conditions such as genotype, developmental stage, and replicate variability. In addition, training the detector does not require an inordinate amount of labeled training examples: detector performance plateaus at eight training images.

Next, we evaluated segmentation accuracy by comparing our implementation of active contours with the more classical method of marker-controlled watershed [46, 47] and a simple baseline method we term “truncated Voronoi” segmentation, which assumes constant radius and non-overlapping cells (see Methods for image pre-processing and implementation of the various algorithms). We hand-constructed segmentations to serve as “ground truth” using Fiji’s “segmentation editor” [14]. Since hand-segmentation of 3D images is an arduous task, we performed this validation on three image stacks, focusing on distal regions comprising a total of 856 cells. To quantify segmentation accuracy, we scored the overlap between an automatically-produced mask and a ground-truth mask (see Additional file 5: Figure S4 for comparison). For each segment, we computed the ratio of the volume of the intersection of the two specified regions to the volume of their union. This ratio, also known as the Jaccard index, has a maximum value of 1 when the segments are identical and penalizes segments returned by the algorithm that are too small or too large. To aggregate accuracy over a whole collection of segmented cells, we first computed an optimal one-to-one matching between the machine and human segments that maximized the overlap between matching segments, and then calculated the average overlap (AO) score averaged across all matched segments.

**Fig. 2:**
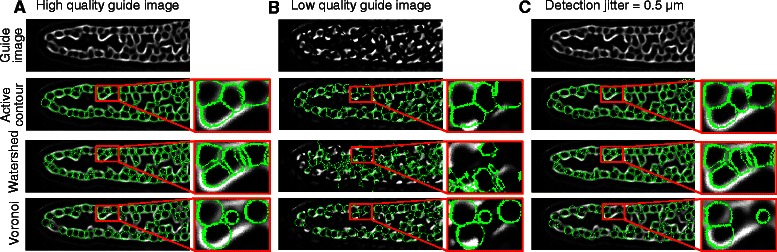
Cell segmentation accuracy. **a** Active contours, marker controlled watershed, and truncated Voronoi all perform well with a high quality guide image and accurately localized cell centers. **b** Marker controlled watershed performs poorly when guide image is low quality. **c** Active contours are more robust to detection noise than truncated Voronoi

Marker-controlled watershed (AO = 0.53 ± 0.06, where the standard deviation corresponds to inter-germline variability), truncated Voronoi (AO = 0.61 ± 0.03), and active contours (AO = 0.62 ± 0.02) performed similarly under ideal conditions with perfectly centered segmentation seeds and clean membrane images (Fig. 2, Additional file 2: Table S3). Since experimental conditions are often less than ideal, we also characterized segmentation accuracy in the presence of a membrane guide image that was artificially degraded to mimic suboptimal staining (see Methods). We found that the segmentation accuracy for marker-controlled watershed decreased drastically when the membrane image was degraded (AO = 0.13 ± 0.01, 75.5 % relative decrease) while active contours were minimally affected (AO = 0.57 ± 0.02, 7.1 % relative decrease). Truncated voronoi does not use the membrane signal and hence is unaffected. To measure the influence of imperfectly localized cell detections, we computed segmentations from marker locations offset by uniform spherical noise of 0.5 μm in radius; this resulted in a 3.3 % relative decrease in AO for active contour segmentation and a larger 8.6 % relative decrease in AO for truncated Voronoi. This noise level roughly matches the statistics of automatic detections, which had an average distance of 0.5 μm from the “true center” (calculated from manually-constructed segmentations). Similarly, if offset noise was increased to 1 μm, the AO of active contour segmentation decreased by 9.6 %, while truncated Voronoi AO decreased by 28.7 %, i.e., nearly three times as much. Altogether, our results demonstrate that marker-controlled watershed is not robust to poor guide image quality and that active contours provide more accurate estimates of cell volume than truncated Voronoi which assumes constant sized cells and does not utilize the guide image (Fig. 2). Thus, our active contour implementation is more appropriate than our marker-controlled watershed or truncated Voronoi implementations for segmentation of our images. We expect this result would likely also hold for other variants of the active contour model such as the explicit surface representations used in [33, 35].

Experts may disagree as to what ground truth should be (see e.g. [48, 49]). To ask how strongly the ground truth we used to quantify performance depends on the particular user that established it, we quantified the agreement between hand-constructed segmentations of an ~80 cell dataset performed independently by three users with Fiji’s “segmentation editor”. Taking one user’s segmentation as ground truth to score agreement with another user, we used the same method as outlined above and derived an AO of 0.64 ± 0.036, precision of 98 % ± 1.5 %, and recall of 97 % ± 3.3 % (Additional file 6: Figure S5). Ground truths derived by different users are thus highly similar. In addition, we note that the level of agreement between users is very similar to agreement of our fully automated procedure with individual users (Additional file 4: Figure S3).

Accurate quantification of fluorescent staining is made difficult in confocal imaging of thick tissues by attenuation loss, which depends on tissue and imaging geometry. To address this, we developed a procedure to identify cells belonging to the top layer of the tissue, which show minimal attenuation (see “Quantification of top-layeredness” in Methods), as well as a normalization procedure, and evaluated the ability of this protocol to produce accurate estimates of DNA content (see “Quantification of DNA content in the *C. elegans* gonadal arms” in Methods). We quantified DNA fluorescence contents in automatically detected cells from gonadal arms that were pulse-fixed with the thymidine analog EdU, which helps identify the phase of the replication cycle in which cells reside at the time of the pulse. We tested accuracy of our estimates of DNA content using the facts that (1) cells at the G1 phase of their cycle have not initiated DNA replication and should thus have minimal content, and (2) that cells in the G2 and M phases have finished replication and should thus have maximal content. As expected, DNA content histograms of cells that were EdU-negative, indicating that they were not replicating their DNA at the time of the pulse-fix and were thus in G1 or in G2/M, displayed characteristic peaks at 2C (minimal content) and 4C (maximal content) with a coefficient of variation of ~20 % (see “EdU content quantification” in Methods; [45]). We verified that our classification of cells as EdU-positive or EdU-negative was accurate; we found specificity and sensitivity of 85 and 88 %, respectively, using manual annotations as “ground truth” (Additional file 2: Table S4). This compares well with the minimal specificities and sensitivities achieved by scoring one user’s EdU status classification using a second user’s classification as ground truth (85 and 92 %, respectively, *n* = 147 cells). Altogether, these results demonstrate that our image analysis pipeline provides accurate fluorescence quantification.

Finally, we tested the accuracy of a cell row counter we developed to assign cells a position along the distal-proximal axis of gonadal arms (counting from the distal end; Fig. 1a; see “Quantification of cell position in the *C. elegans* gonadal arms” in Methods). Counting in cell rows rather than in “physical distance” is useful for comparison with reports in the literature, which conventionally use this unit of measurement. We compared measurements of MZ length performed using our cell row counter against manual measurements. The average deviation of automatic cell row measurements from manual measurements was 9.4 %, and we observed strong correlation between measurements (*R* ^*2*^ = 0.72; Additional file 7: Figure S6). This compares well with the average deviation between MZ lengths performed independently by two users (12 %, *n* = 9 MZs), indicating that our cell row counter can adequately substitute for human scoring.

### Comparison to other software

To help place our technique on the fast-evolving landscape of image segmentation software, we evaluated the performance of well-established solutions on the same hand-segmented stacks we used to assay our pipeline. We initially tested a large number of possibilities (including Icy [11], Farsight [7], Ilastik [9], Fiji and Trainable Weka [14], Tango [15], BioImageXD [12], and V3D [8, 50]) and focused on four that appeared particularly promising for our image data: Imaris (Bitplane), Vaa3D Gradient Vector Field (GVF) plugin [17], Ilastik [9], and MINS [16] (see “Comparison to other software” in Methods for setup details). Our pipeline had substantially lower false positive and false negative rates than Imaris, Vaa3D, Ilastik, and MINS (precision and recall 97 % ± 0.73 % and 93 % ± 5.6 % vs e.g. 90 % ± 1.4 % and 77 % ± 3.4 % for MINS or 92 % ± 1.5 % and 72 % ± 3.7 % for Vaa3D; Additional file 2: Table S5; see Additional file 8: Figure S7 for example Ilastik and MINS output, and Methods for derivation details), and also higher segmentation accuracy for cells that were correctly detected (Additional file 2: Table S5; AO = 0.60 ± 0.015 vs 0.37 ± 0.047 for the runner-up, Imaris).

To verify that our comparison was robust to variations between experts providing ground truth, we used the same dataset introduced above consisting of ~80 cell segmentations hand-constructed by three users independently. This set of cells was located at the distal end of a mitotic zone, because these cells are more densely packed than proximal cells and are thus expected to provide stronger discrimination between different techniques. Using this set of cells actually increased AO for Imaris (to 0.54 ± 0.039), but Parismi still had higher AO (0.61 ± 0.038), precision (97 % ± 2.3 %), and recall (100 % ± 0.8 %) than Imaris, Vaa3D, Ilastik and MINS (runner-ups in each category scored at 0.54 ± 0.039, 96 % ± 0 %, and 85 % ± 2.4 %, respectively; Additional file 2: Table S6). We emphasize that these results are specific to our dataset — but in any case they demonstrate that our pipeline is an appropriate tool in this setting.

Parismi’s graphical user interface (GUI) makes it possible for a user to review and correct detections spending about 15 min per gonad mitotic zone. Using Fiji’s “segmentation editor”, user estimates of time spent to hand construct segmentations range from 8 h per mitotic zone (using 3D interpolation) to 14 h (without using 3D interpolation). Importantly, time curating Parismi detections is mostly spent examining images rather than actively drawing cell outlines — which is particularly taxing, both mentally and physically, when done on a large scale. In addition, Parismi enables fast human annotation of detected cells with custom labels (in as little as a single click per cell or per group of cells, for a pre-selected label). Overall, Parismi thus enables better use of human time, with interactions that are targeted at fixing specific issues in segmentations rather than creating them from scratch.

### Spatial distribution of cell cycle phase indices in the *C. elegans* germ line

**Fig. 3:**
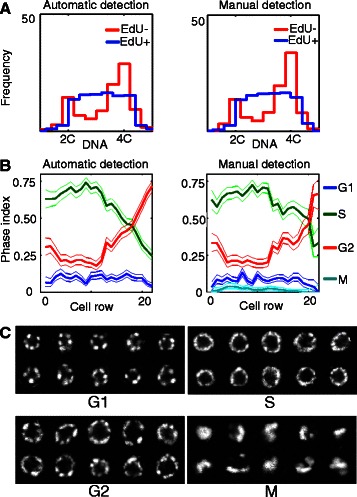
Cell cycle phase studies of the *C. elegans* germ line using spatial cytometry. **a** DNA content histogram of EdU^−^ (red) and EdU^+^ (blue) germ cells computed automatically (left) and with manual seeding (right). **b** Cell phase indices in the germ line MZ computed automatically (left) or with manually-curated seeding (right). **c** Nuclear morphology of G1, S, G2, and M-phase cells

To validate our approach in a way that goes beyond the benchmarks provided above, we decided to compare results derived from our spatial cytometry pipeline — run with fully-automatic cell detection or with manually-curated cell centers — to results from the literature derived using classical techniques. We relied on 48 *C. elegans* EdU pulsed-fixed gonadal arms containing 12,997 segmented MZ cells, from which we derived cellular DNA and EdU contents that we used to categorize cells as being in G1, S, or G2 phase (in this approach we ignored M-phase cells, which are present at only ~3 %). Results derived from either fully-automatic cell detection or manually-curated centers were virtually identical (Fig. 3a-b; see also [45] for manual results, with the difference that they did not rely on the cell row counter reported here). Defining cell cycle phase “index” as the proportion of cells found at that particular phase, we observed a decrease in G2 index along the first four cell rows of the gonadal arms, with the G2 index of the first cell row being significantly higher than that of the fourth cell row (p < 0.01, categorical chi-square test; Fig. 3b; this result was unchanged when manually annotating M-phase cells). This finding is of interest because it shows distinctive cell-cycle behavior of stem cells located at the distal end of the MZ, at the G2 cell cycle phase. While it was previously observed that M-phase index differs along the distal-proximal axis of the gonadal arm [51], our approach makes it possible to examine the behavior of stem cells at all other phases of the cell cycle — which together account for ~97 % of cell cycle time.

### Morphological predictors of cell cycle phase in the *C. elegans* germ line

**Fig. 4:**
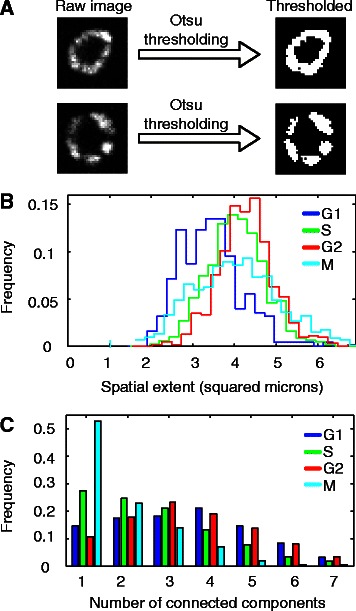
Morphological features of G1, S, G2, and M phase of the cell cycle. **a** Thresholding of raw cell images via Otsu's method to partition DNA signal into foreground and background pixels. **b** Spatial extent of DNA fluorescence, assayed by counting the number of foreground pixels in thresholded images. On average, G1 is smaller than S is smaller than G2. See Additional file 2: Tables S9, S10 for statistical comparisons. **c** Spottiness of DNA morphology, assayed by counting the number of connected components in thresholded images. On average, G1 and G2 phase cells have more connected components than S phase cells, which have more connected components than M phase cells. See Additional file 2: Tables S7, S8 for statistical comparisons

As a second step in deriving new results based on segmentations, we asked whether DNA morphology changes with phase of the cell cycle. Although M-phase and its sub-phases have characteristic morphologies that can be classified using machine learning approaches [52, 53], the other phases G1, S, and G2 superficially appear similar to one another. Previous studies have suggested that cell images carry information enabling computer analysis to distinguish between cell types (e.g. [54]), and in particular that a relationship exists between chromatin texture and cell cycle phase [55, 56]. Our observations made using mosaics of segmented and classified cells (Additional file 9: Data 1) suggested that the DNA of G1 phase cells tends to have a punctate morphology with approximately 5–6 puncta per cell; S phase DNA has a smooth morphology without readily visible puncta; and G2 phase DNA has a punctate morphology with a variable number of puncta (Fig. 3c). In addition, the area covered by DNA in G2 phase cells appeared larger than that in S-phase cells, which in turn appeared larger than that in G1-phase cells. In order to place these observations on a more quantitative footing, we chose a 2D slice in the middle of each cell, thresholded the DNA image from each of those slices using Otsu’s method [57], and measured the total number of connected components (i.e., the number of spots) in segmented DNA as well as the area (i.e., the spatial extent) of the segmented DNA. We found that the average number of spots in G1 or G2 phase nuclei is larger than that observed in S-phase nuclei (p < 1e–12, Bonferroni corrected rank-sum test; see Fig. 4, Additional file 2: Tables S7–S8), and that the average spatial extent of DNA fluorescence in G1 phase nuclei is smaller than that in S-phase nuclei, which in turn is smaller than that in G2-phase nuclei (p < 2e–14, Bonferroni corrected rank-sum test; see Fig. 4, Additional file 2: Tables S9–S10).

Having established that morphological differences exist between the nuclei of G1-, S-, and G2-phase cells, we asked whether these differences could provide a basis for cell phase classification. We extracted the following features from segmented nuclei: (1) the number of connected components of the thresholded foreground mask, (2) the total number of pixels composing the thresholded foreground mask, and (3) Haar-like features [58]. We used these features to train SVM classifiers (see “Morphological classification of cell phase” in Methods), whose mean sensitivity and specificity exceeded 0.66 for all classifiers (Additional file 2: Table S11). Thus, segmentation of individual cells shows that DNA morphology carries substantial information about cell cycle phase. A crucial advantage of using this morphological information is that it is acquired as a matter of course in most imaging experiments, and does not require fluorescent transgene expression or live imaging that facilitate cell cycle phase identification (e.g. [59–64]) but limit the kind of tissue that can be imaged, the strains that can be used, and the number of imaging channels that are available for readouts unrelated to the cell cycle. Future work will focus on improving classifier performance, using an extended set of features and more powerful classification techniques, which will enable practical applications.

### Cell cycle arrest upon starvation in the *C. elegans* germ line

**Fig. 5:**
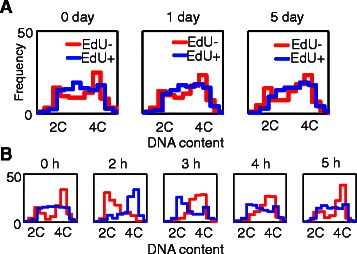
Cell cycle properties of starved worms. **a** DNA content histograms of EdU^−^ and EdU^+^ cells remain constant over the course of 5 days when adults are starved, indicating that little to no cell cycle progression occurred from the onset of starvation. **b** For comparison, there is clear cell cycle progression over 5 h in well-fed worms

As a third application of segmentation, we asked how *C. elegans* germ cells respond to worm starvation, which is expected to occur frequently in the wild [65]. Although the germ line is known to undergo dramatic cell death or regeneration upon changes in nutritional status [66], and larval germ cells are known to arrest in G2 in starved larvae [67, 68], the kinetics of cell cycle response to food removal remain uncharacterized in adults. To ask whether cells stop at a particular point of the cell cycle, we tracked cell cycle progression of labeled and unlabelled cells in germ lines pulsed with EdU and chased over a five-day starvation period. We observed little to no cell cycle progression (Fig. 5a) in two independent experimental repeats that included a total of 20,022 MZ cells in 73 gonadal arms. This unexpected result suggests that there are a large number of points along their cycle at which cells can pause in response to food removal, at least in adult nematodes.

### Pre-implantation mouse embryos

We next turned to datasets from pre-implantation mouse embryos and the mouse olfactory placode. First, we asked whether our active contour implementation generalized to images from these systems, whose appearance is substantially different from that of *C. elegans* gonadal arms. We found that, with some adjustments to the image pre-processing steps applied prior to running active contours (see “Preprocessing of mouse pre-implantation embryo guide images” Methods), cells from these three sources could be suitably segmented (Fig. 1d-e). Second, we evaluated automatic detection accuracy in pre-implantation mouse embryos (see Methods). We found detection performance to be comparable to that for worm germ cells: the detector’s AP was 96.8 %, and the precision-recall curve shows that, with an appropriate threshold, we detect more than 80 % of cells without a single false positive (Additional file 4: Figure S3).

**Fig. 6:**
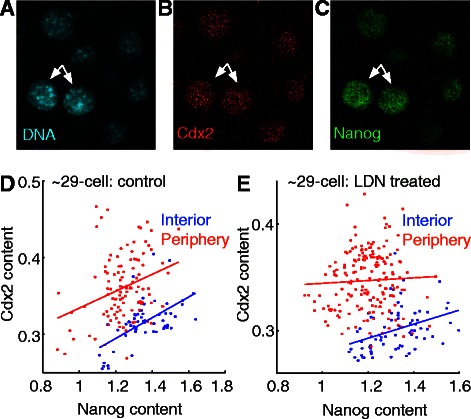
Relationship between Nanog and Cdx2 contents in preimplantation mouse embryos. **a**-**c** Mouse embryo stained for DNA (**a**) Cdx2 (**b**) and Nanog (**c**; white arrows point to the same two cells in each panel). **d** Control embryos cultured for 12 h (28.5 ± 6.8 cells per embryo, *n* = 8) show a positive relationship between Nanog and Cdx2 (interior linear fit slope 0.13, 95 % CI = [0.08, 0.18]; periphery linear fit slope 0.11, 95 % CI = [0.05, 0.17]). **e** Embryos cultured for 24 h with BMP signaling inhibitor LDN (29.4 ± 10.2 cells per embryo, *n* = 12) do not show a positive relationship between Nanog and Cdx2 content (interior linear fit slope 0.07, 95 % CI = [0.03, 0.10]; periphery linear fit slope 0.01, 95 % CI = [−0.03, 0.05])

We next asked whether we could derive new insights in regulation of cell differentiation in pre-implantation mouse embryos (see [69] for another application). We quantified expression of Cdx2 and Nanog, two antagonistic regulators involved in the early differentiation decision made in the embryo that guides the establishment of an embryonic stem cell-like population [70, 71]. Despite being antagonistic, these factors are co-expressed early during embryonic development ([71]; Fig. 6a–c); such paradoxical early co-expression is also true of a number of other antagonistic gene groups that mediate cell fate decisions, and models have been proposed to account for initial co-expression or concomitant upregulation of antagonistic factors [72–74]. To further assess the relationship between Cdx2 and Nanog in different cell sub-populations in the pre-implantation mouse embryo, we used a recently-published dataset in which we had annotated cells as being either on the “interior” of the embryo or on the “periphery” of the embryo and quantified the levels of Cdx2 and Nanog in each subpopulation [69]. As expected, cells on the periphery of the embryo had higher Cdx2 content and lower Nanog content than cells on the interior (Fig. 6d). Interestingly, we observed that despite the Nanog/Cdx2 antagonism both inner and outer cell populations show a significant positive correlation between Cdx2 and Nanog expression levels at the ~29 cell stage (Fig. 6c). Surprisingly, however, the positive correlation was removed specifically in the outer cells upon chemical inhibition of BMP signaling, which we recently showed to be active in early embryonic development (Fig. 6e; [69]). These results suggest that BMP signaling may play a context-dependent role in the regulatory interactions between Nanog and Cdx2 or their upstream controls; this intriguing context-dependence would have been obscured had we evaluated only average expression levels across all cell sub-populations. These findings further emphasize the utility of segmentation methods in understanding complex regulatory networks that underlie cell differentiation.

### Mouse olfactory placode

**Fig. 7:**
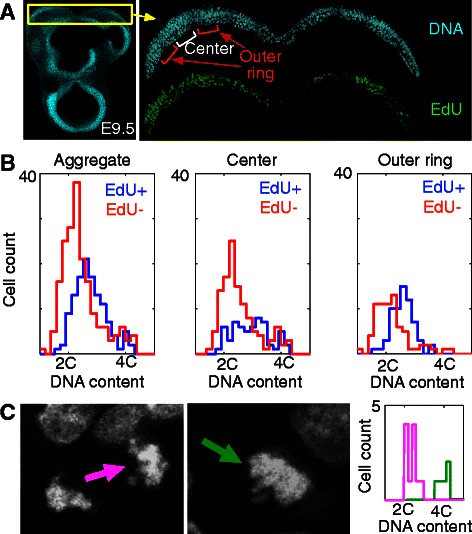
Differences in cell phase indices along the medial-lateral axis of the mouse olfactory epithelium. **a** *Left*: A transverse vibratome section of an E9.5 embryo including the two olfactory placodes (yellow box). *Right*: High magnification image of the placodes stained for DNA (cyan) and EdU (green). Placodes were divided into “center” and “outer ring” regions for analysis. **b** DNA content histograms of EdU^+^ (red) and EdU^−^ (blue) cells in center and outer ring regions. **c** During cell detection, M-phase cells in the process of cytokinesis were segmented as two separate cells and were annotated as being a “half cell”. Conversely, M-phase cells before cytokinesis were annotated as being a “full cell”. DNA content histograms of “half” (magenta) and “full” (green) cells are appropriately centered at 2C and 4C, respectively

Finally, we asked whether cell segmentation could allow us to make new findings on cell cycle behavior during embryonic development, using mouse olfactory placode as a model system (Fig. 7a). The olfactory placode is a thickened region of head ectoderm that invaginates into developing head mesenchyme to form the olfactory mucosa, a highly-branched mucosa whose epithelial lining contains the primary sensory neurons that subserve the sense of smell [75]. Questions remain about the forces that drive the early phases of invagination in this and other ectodermal placodes of the head. A number of possible mechanisms have been proposed for such morphogenetic changes, including local modulation of cell proliferation rates [76]. We used Parismi to ask whether we could detect different cell cycle behavior in sub-regions of olfactory placodes, which we called center and outer “rings” of each placode based on known patterns of gene expression and cell differentiation state [75, 77]. We developed a staining protocol for the placode that relies on thick, minimally processed vibratome sections, because traditional frozen sections did not produce tissue of sufficient quality for our analysis (see Additional file 10: Figure S8 for a comparison, and “Mouse olfactory placode image acquisition” in Methods). We processed and imaged placodes from the developing heads of E9.5 mouse embryos, and categorized cells as G1-, S-, G2- (based on DNA content and EdU content; see “Quantification of DNA content in the mouse olfactory epithelium” in Methods), or M-phase (using manual annotations based on DNA morphology). S-phase index was higher in the outer rings than in the center rings of these placodes (*p* < 0.0062, categorical chi-square; Fig. 7b). There was also a significant change of overall cell cycle phase distribution (*p* = 0.037, categorical chi-square test). The increased S-phase index of outer ring placode cells is particularly interesting because this is the region in which Sox2- and Fgf8-expressing stem cells of the early olfactory epithelium are found in highest number [77], suggesting that proliferation of these stem cells may be important for driving early morphogenesis of the olfactory epithelium.

## Conclusions

The biological results we report here could not have been derived without distinguishing between subpopulations of cells in the tissues that we studied. In some model systems cell types could perhaps be distinguished using cell-type specific markers and analysis of dissociated cells, but relevant markers are not always known or available. Some, but not all, of the cell cycle results we report could also conceivably have been derived using “double labeling”, which relies on the use of two cell cycle phase labels (e.g. BrdU and EdU) applied in succession, and binary classification of cells as positive or negative for each of the two labels — and thus does not require quantification of fluorescence contents (e.g. [78, 79]). But such double labeling requires extra experimental manipulation and imaging of two fluorescence channels in addition to the one normally used for DNA visualization. Limitations on multiplexing capabilities make it advantageous to rely on imaging of DNA and a single cell cycle label to 1) identify cell types, relying in part on position in the tissue and morphology, and 2) to infer cell cycle characteristics; using a single label frees fluorescence channels to quantify other properties of interest on a cell-by-cell basis.

Overall, we have provided a detailed methodology to answer different kinds of questions within the context of the *C. elegans* gonadal arms, the mouse pre-implantation embryo, and the mouse olfactory epithelium. This methodology relies on the use of 3D imaging of cells in their native setting, followed by quantification of fluorescence content on a cell by cell basis. Future developments of direct interest to biologists working on these model systems or similar systems may include making more extensive use of morphological information to increase accuracy of cell cycle phase classification and to identify cell types without the use of specific markers.

The software pipeline developed for the applications in this report has been made freely available, along with image datasets and curated gold-standard annotations for these images. While researchers working on the same or similar model systems may prefer to utilize other software ecosystems (such as Fiji, Vaa3D, BioimageXD, Icy, or other workflow architectures that offer plugins to perform similar detection and segmentation tasks), we expect that the system reported here will provide a useful guide of overall methodology and baseline measures of performance. In particular, unlike workflows that derive from the long tradition of adaptive thresholding followed by morphological processing to identify individual objects, the two-phase strategy utilized here of detection followed by seeded segmentation appears to be quite robust, providing a modular workflow that allows easy injection of “high level” manual curation by adding or removing detections with a single click prior to segmentation, rather than tedious editing the final segmentation output at the pixel level.

## Methods

### Datasets

Datasets supporting results of this article are available at http://cinquin.org.uk/static/Parismi_datasets.tgz. The datasets contain original images for ~45,000 worm germ cells, Parisimi segmentation pipelines, as well as segmentation results stored both as Google Protobuf files (the format is specified at https://github.com/cinquin/parismi/blob/master/A0PipeLine_Manager/src/protobuf_seginfo_storage.proto) and as TIFF files in which pixel value denotes cell index. Parismi source code is available at https://github.com/cinquin/parismi/

### *C. elegans* gonadal arm image acquisition

Young adult worms (staged 24 h after the last larval stage L4) were pulsed with the thymidine analogue EdU (Invitrogen Carlsbad, CA USA) for 30 min to label cells in S-phase, fixed, and processed as described [80]. For starvation experiments, L4 + 1 day virgin *fog-2* females were pulsed with EdU for 30 min and immediately transferred from *E. coli* plates, washed multiple times and starved in S-medium for 5 days. DAPI was used to label DNA and an α-PH3 antibody to label M-phase cells. Gonadal arms were imaged at 0.3 μm z-intervals with Zeiss LSM 710 or 780 confocal microscopes. Gonadal arms with abnormal morphology (*n* = 2/159) were excluded from the cell cycle analysis.

### Mouse olfactory placode image acquisition

Embryos were staged by designating mid-day of vaginal plug detection day as embryonic day 0.5 (E0.5). E9.5 embryos were obtained by crossing CD1 mice (Charles River Laboratories, Wilmington, MA USA). For pulse-fix analysis of EdU incorporation, EdU was injected intraperitoneally into pregnant dams (12.5 μg/gm body weight) and embryos were collected 30 min later. Dissected tissues were fixed with 4 % paraformaldehyde in PBS (Sigma-Aldrich, St. Louis, MO), embedded in 7 % LMP agarose (Fisher BP165-25) and vibratome sectioned (100 μm thickness). To create a guide image for active contours, we stained with rabbit polyclonal anti-cadherin (1:500; Thermo, Waltham, MA USA: clone # PA5-19479), detected with Cy2-conjugated donkey anti-rabbit-IgG (1:100; Jackson ImmunoResearch, West Grove, PA, USA). After the secondary immunostaining reaction, tissue was processed with the EdU Click-iT Kit (Invitrogen). DNA was detected with bisBenzamide H 33342 trihydrochloride (10 μg/ml in PBS). Samples were mounted on slides in Aqua-mount (Thermo Scientific 13800) prior to imaging at 0.3 μm z intervals with Zeiss LSM 710 or 780 confocal microscopes. Samples without M-phase cells (required for DNA normalization) were excluded from analysis (*n* = 4/6 samples covering a total of two OE invaginations were used).

Pre-implantation mouse embryo images were acquired as previously described [69].

All protocols for animal use were approved by the Institutional Animal Care and Use Committee of the University of California, Irvine.

### Overall software organization

Parismi is designed as a highly-modular pipeline with a Java scheduler, plugins written in Java, C++, or Matlab that perform image segmentation and quantification. The plugins are decoupled from the input/output code and from the graphical user interface, in part through the use of parameter injection with Java field annotations, and have parameters serialized in a way that allows for backward-compatible changes to the set of plugin parameters. This ensures high reusability of the code in future projects, and high reusability of archived pipelines designed for and run using older plugin versions. The pipeline scheduler can automatically parallelize plugins (e.g. a plugin that works on 2D image planes can be made to run automatically in parallel on 4D stacks), which minimizes the amount of coding and code repetition. Many plugins (such as the active contour plugin) are in addition internally multithreaded. Depending on the dataset Parismi can thus take full advantage of a large number of processor cores (up to 64 in our usage). Run time for *C. elegans* MZ stacks is about 4 min for cell detection, and about 7 min for downstream analysis on a 64-core machine.

Parismi can run in a fully automated mode on multiple datasets, either from the GUI or from the command line — in a way that relies on Makefiles to minimize re-computation after input or parameter changes [81]. It can also run in an interactive mode as an ImageJ [13] plugin that allows for Parismi plugin parameters to be adjusted on an image-by-image basis with live updates and graphical display of quantification results; Parismi makes it straightforward to quickly annotate 3D image structures by clicking and dragging on any of three orthogonal views, and to move from one dataset to the next without manual closing and opening operations (see Additional file 11: Figure S9 for GUI screenshot). The pipeline can be re-run from the original set of images using the record of edited cells and of any adjusted plugin parameters. A more detailed operation overview is available (https://github.com/cinquin/parismi/raw/master/Parismi_operation_overview.pdf).

### Cell detection

We trained an automatic cell detector that predicts cell center locations from DNA-stained image stacks by classifying each sub-window of the stack as either containing a cell center or not. Positive example sub-windows were specified by hand-clicking cell centers, and negative examples automatically extracted from locations farther than one cell radius from all positive labels.

We extracted image features from the training set using 2D windows taken along the xy and xz planes running through detection centers. From the xy slice we computed two features: average pixel brightness of the detection window, and a histogram of oriented gradients (HOG features) computed over a grid of non-overlapping sub-windows ([82]; Additional file 3: Figure S2). Within each sub-window, the image gradient was estimated at each pixel and binned into one of 18 orientations. These histograms were normalized and the normalization factor along with the normalization of neighboring bins were stored as additional features. The same HOG features were computed for the xz slice. Since there was no *a priori* favored image orientation, the features were symmetrized left-right and top-bottom for positive training examples. Overall, 2,147 cells across 6 gonadal arms were used for training.

We trained a support vector machine (SVM) classifier to distinguish positive detections from negative detections in the training set. A given feature vector corresponds to a cell center if

$$ {w}^Tv>\tau $$

where w is a weighted vector learned by the SVM, v is a feature vector, and τ is a tunable parameter. A lower value of τ yields more cell detections at the cost of more false positives. Since there are millions of negative detections in our training set, we used an iterative training algorithm to decrease run time and memory requirements. This iterative algorithm trains the detector with a subset of negative examples, searches for additional high-scoring negative examples, adds them to the training set, and retrains the classifier. This iterative approach of hard negative mining is mathematically equivalent to performing training on the set of negative detections.

To make the training algorithm robust to potential errors in the localization of cell centers by manual labeling, we performed latent estimation of the “true” cell center for each positive training example [83]. Briefly, once a detector had been trained, we ran the detector on the positive training data and re-estimated the center of each cell as the maxima of the detector response within a small radius of the original ground-truth detection. The detector was then re-trained with these updated set of positive locations.

The final step in automatic detection applies the SVM classifier to all sub windows in a given DNA stained image. To accurately handle natural variation in cell sizes, this detection process was carried out multiple times on scaled versions of the original image stack (scale factor ranging from 0.7–1.5 for MZ stacks). Since a given cell may produce multiple positive detections in slightly offset sub-windows, we suppressed detections which overlapped with any higher-scoring detection within one cell radius. Overall, the testing dataset size was 3579 cells across 10 gonadal arms.

For automatic detection of pre-implantation mouse embryo nuclei, we used 16 image stacks containing 484 cells in total (15–56 per stack). We used a single stack containing 24 cells for training, keeping the detection parameters used for *C. elegans* gonadal arms. Of the remaining 15 stacks, we used 7 as a validation set, to find the scale range and detection suppression radius that maximized average precision, and 8 as a test set; detections were considered to be correct if they were within a distance 0.35 * d from the manually-annotated cell center, where d is the cell diameter (defined as the average of manually-determined cell width and cell height). Average precision, defined as average precision over all recall values, is the same as the area under the precision-recall curve (Additional file 4: Figure S3).

For results shown in Figs. 6 and 7, cell centers in images of the mouse OE and mouse embryos were curated manually.

### Preprocessing of *C. elegans* gonadal arm and mouse olfactory epithelium guide images

We preprocessed membrane images to facilitate subsequent image segmentation steps. First, we removed low frequency noise from the membrane image by “sharpening” the image. We normalized pixels to the average pixel value in a surrounding 2D sliding square parallel to the xy-plane; we chose the size of the sliding square to be the average cell diameter; this normalization made fluorescence contents even across the z-axis. Second, we removed sharp discontinuities and high frequency noise by blurring the sharpened image. We set the standard deviation of the Gaussian kernel to the membrane thickness. Third, we enhanced “sheet-like” structures using a principal curvature approach [84]. For each pixel in the blurred image I, we calculated the Hessian matrix H:

$$ H\left(x,y,z\right)=\left[\begin{array}{ccc}\hfill {I}_{xx}\hfill & \hfill {I}_{xy}\hfill & \hfill {I}_{xz}\hfill \\ {}\hfill {I}_{yx}\hfill & \hfill {I}_{yy}\hfill & \hfill {I}_{yz}\hfill \\ {}\hfill {I}_{zx}\hfill & \hfill {I}_{zy}\hfill & \hfill {I}_{zz}\hfill \end{array}\right] $$

with ordered eigenvalues d_1_(x,y,z), d_2_(x,y,z), d_3_(x,y,z). Then, we enhanced sheet-like structures by computing the intermediate image I’:

$$ I\hbox{'}\left(x,y,z\right)=-{d}_1{e}^{-{\left(\frac{d_2}{2{d}_1}\right)}^2}{e}^{-{\left(\frac{d_3}{2{d}_1}\right)}^2} $$

Finally, we generated the final preprocessed image by removing sharp discontinuities through blurring, with the standard deviation of the Gaussian kernel again set to the membrane thickness.

### Preprocessing of mouse pre-implantation embryo guide images

For mouse pre-implantation embryos we used the DNA channel as a guide image for segmentation. We preprocessed the images as follows. First, we thresholded the DNA image (D) using an adaptive algorithm. Let *p* = {x,y,z} be a given cell detection point, and let {p_i_} be a set of cell detections. For a small window around a given p_i_, we calculated the mean DNA pixel value m(x_i_,y_i_,z_i_). Then, we fit coefficients c_1_,c_2_,c_3_,c_4_ to the model m(x,y,z) = exp(c_1_z) (c_2_x + c_3_y + c_4_). We calculated an adaptive threshold t(x,y,z):

$$ t\left(x,y,z\right)=k{e}^{c_1z}\left({c}_2x+{c}_3y+{c}_4\right) $$

and applied this threshold to the DNA image. We chose k heuristically; *k* = 1/3 worked well in practice. Second, we removed removed high frequency noise from the thresholded image through median filtering. Third, we inverted the image. Finally, we removed sharp discontinuities via blurring, yielding the final preprocessed guide image for active contours.

### Artificial degradation of preprocessed guide images

A degraded preprocessed guide image was generated for segmentation benchmarking using the following procedure:

1. Let *I* denote the original preprocessed membrane image. An image kernel *K* was generated by cropping a 108 x 108 x 40 section of *I*.
2. The kernel was inverted and thresholded so that no pixel values fell above 0.85. Then, the kernel was scaled so that all pixel values fall between [0,1].
3. The kernel was tiled to form a new image the same size as *I*.
4. The degraded preprocessed image was generated by multiplying *I* and *K*.

### Active contour segmentation

Implicit active contours have been used extensively in biological image analysis [29–35]. We use an implicit formulation in terms of level sets first described in [85]. Active contours are model-based and work well even in the presence of a poor guide image. Since *C. elegans* germ cells are roughly spherical and uniform in size, active contours represent a good choice for segmenting them. Consider the partial differential equation:

$$ \frac{\partial \phi }{\partial t}=\mathit{\mathsf{g}}\left\Vert \nabla \phi \right\Vert \left(1-{c}_1k\right)+{c}_2\nabla \mathit{\mathsf{g}}\cdot \nabla \phi $$

used to update active contours. Here, g is the inverted preprocessed guide image, ɸ is a higher dimensional function that embeds segmentation mask composed of points where ɸ(x,y,z) < 0, and *k* is the mean curvature:

$$ k=\nabla \cdot \left(\frac{\nabla \cdot \phi }{\left\Vert \nabla \phi \right\Vert}\right) $$

used to enforce smooth segmentation borders. ɸ is initialized at cell detection points and then active contours are run in two steps. The first step of active contours is conservative; masks stop short of boundaries in the guide image. This is achieved with c_2_ < 0 so that contours are pushed backwards as they approach inner edges. The second step of active contours refines the masks so that they stop on the boundaries of the guide image. This is achieved with c_2_ > 0 so that the contour is pulled forward as it approaches an inner edge, then pushed back as it approaches an outer edge. In order to prevent overlapping segmentation masks, we set dɸ(x,y,z)/dt = 0 when two different masks “collide”, i.e., when ɸ_q_(x, y, z) < 0 and ɸ_p_(x, y, z) < 0 for any two q ≠ p where ɸ_q_ is the segmentation mask for cell q. Hence, all contours are blocked at any position where collision has occurred (a future improvement would be to include a more flexible penalty term in the energy [30, 31], which would allow the constraints to remain active even after collision has occurred). Typical active contour parameter values are c_1_ = 0.1 and c_2_ = −1 for the first step of active contours, and c1 = 0.1 and c2 = 1 for the second step. The stopping criterion for contour evolution is a set run time (for step 1: 650 time steps of 0.15 each, and for step 2: 300 time steps of 0.15 each).

In order to decrease run-time, we ran individual ɸ corresponding to individual cells in cropped sub-windows. In addition, we computed updates to ɸ using the narrow band level set method [30, 31, 86]. Finally, we note that while we chose the implicit representation for ease of implementation, recent approaches based on explicit surface representation [33, 35] are faster and more memory efficient.

### Truncated Voronoi

For a set of cell detections *p* = {p_1_,p_2_,…,.p_n_}, consider the following:

1. The Voronoi diagram V consisting of Voronoi polygons so that V = {v_1_, v_2_, …, v_n_}
2. The set of points c_i_ within radius r of point p_i_ so that C = {c_1_, c_2_, …, c_n_}

We computed the truncated Voronoi segmentation S as segmentation masks s_i_ that consist of the intersection of points between v_i_ and c_i_

$$ \mathit{\mathsf{S}}=\left\{{\mathit{\mathsf{s}}}_{\mathit{\mathsf{1}}},{\mathit{\mathsf{s}}}_{\mathit{\mathsf{2}}}, \dots, {\mathit{\mathsf{s}}}_{\mathit{\mathsf{n}}}\right\},{\mathit{\mathsf{s}}}_{\mathit{\mathsf{i}}}={\mathit{\mathsf{v}}}_{\mathit{\mathsf{i}}}\cap {\mathit{\mathsf{c}}}_{\mathit{\mathsf{i}}} $$

Note that the result of truncated Voronoi only depends on input cell detections, and not on the preprocessed guide image. This provides a naïve baseline against which more elaborate approaches can be compared.

### Marker-controlled watershed

A detailed description of marker-controlled watershed can be found in [47]. In brief, let I be the guide image used to define segmentation boundaries and let p be a set of cell detections. We imposed local minima at I(p) via morphological reconstruction. Then, we ran a watershed transform on I in order to generate segmentation masks. We thresholded segmentation masks based on size in order to remove gross mis-segmentations.

### Comparison to other software

We ran the Vaa3D GVF plugin using 5 diffusion iterations, a fusion threshold of 1, and a minimum region size of 999. We performed an extensive coordinate-wise search over parameter settings, choosing these parameter settings which we found yielded the best segmentation performance. We ran the Imaris v8.1.2 cell detection module both with default parameters and with hand-adjusted parameters, and report best results; scoring of the exported segmentations required ad-hoc removal of segment borders and anti-aliasing artifacts. We ran Ilastik v1.1 with hysteresis thresholding (low and high set to 0.20 and 0.80, respectively), using ~230 brush strokes to provide examples of background and foreground pixels, and tried a number of feature combinations: fine-scale features only, fine- and medium-scale features, medium- and coarse-scale features, coarse-scale features only, and all features. Overall the choice of feature combinations had a minor effect, but coarser-scale features (sigma = 5.0px, sigma = 10.0px) seemed to work best. The main limitation of Ilastik was its difficulty in separating cells that are tightly packed together and thus not neatly separated by pixels it is trained to recognize as background (Additional file 8: Figure S7A). We ran MINS v1.3 setting the noise level to 3 and using the default smoothing settings. Although MINS performed well in cell detection, its segmentations did not adhere as closely to cell boundaries as manual and Parismi segmentations did (Additional file 8: Figure S7B-E), which explains its low AO score. Note that because of RAM requirements MINS and Ilastik were run on cropped images, which may increase their apparent accuracy because it eliminates opportunities for false positives outside the crop region.

### Quantification of top-layeredness

“Top-layeredness” is a metric we used to identify cells on the “top layer” of the gonadal arms (Additional file 12: Figure S10). During imaging, these cells have direct line of sight to the microscope objective and thus exhibit minimal attenuation along the z-axis. We defined the top-layeredness θ of a cell to be the fraction of its segmentation mask that is unobscured in the z-projection over all cell segmentation masks. Thus, θ = 0 means that a cell is completely obscured from the path of the microscope objective, while θ = 1 means that a cell has direct line of sight to the microscope objective. In practice, θ > 0.1 was a good threshold to define cells on the top layer of the gonadal arm. Due to the relatively small number of cells per image, top-layer thresholding was neither applied to mouse olfactory epithelium images nor to mouse embryo images.

### Quantification of cell position in the *C. elegans* gonadal arms

We computed cell spatial position in two ways:

1. Based on “geodesic distance”. We fit a principal curve to cell detection points using the algorithm detailed in [87]. We then computed the distance of each cell to the distal end of the gonadal arm along the principal curve (distal ends were manually annotated).
2. Based on cell row distance. We generated a connectivity map between germ cells based on touching segmentation masks. We then computed the minimal path of a given cell to the distal end of the gonadal arm via Dijkstra’s algorithm [88] (Additional file 7: Figure S6A).

### Quantification of DNA content in the *C. elegans* gonadal arms

The naive way to calculate cell DNA content would be to simply sum pixels in a given segmentation mask. However, appreciable fluorescence attenuation often occurs along the distal-proximal axis and z-axis of the gonadal arm, which can introduce bias into spatial cell cycle studies. In addition, it is not straightforward to aggregate data in different experimental replicates since staining efficiency is sometimes variable. We corrected for these artifacts using the following normalization procedure:

1. We filtered segmented cells to only keep those in the “top layer”, which minimized artifactual variations in DNA content due to fluorescence attenuation along the z axis.
2. For each segmentation mask, we computed the raw DNA content (sum over all DNA pixel values inside the mask) and the 95 % DNA content percentile (95 % percentile of DNA pixel values inside the mask).
3. We fit a cubic spline to the empirical distribution of 95 % DNA content percentiles as of function geodesic distances, on a gonad-by-gonad basis. We normalized raw DNA contents against this spline to derive spline-normalized DNA contents. This step reduces potential bias from fluorescence attenuation along the distal-proximal axis of the gonadal arms.
4. Cellular data was binned by spatial position. The 10th and 85th percentile of spline-normalized DNA contents in each bin was normalized to 2C and 4C DNA content, respectively. A bin size of four cell rows was used. This step allows us to aggregate data across germ lines by assuming each spatial bin contains the same proportion of G1/S/G2/M-phase cells across germ lines.

### Quantification of DNA content in the mouse olfactory epithelium

We corrected for fluorescence fluctuation along the long, medial-lateral axis of the OE (which corresponds to the x-axis in our images) by fitting a second order polynomial to the 90 % percentile pixel intensity in each x-slice, then normalizing against this polynomial. We corrected for z-attenuation by fitting a first order polynomial to the 90 % percentile pixel intensity in the middle 25 z-slices of each image, then normalizing against this polynomial. We used the middle 25 z-slices because cells were evenly distributed in this region.

We measured DNA content for each cell by summing the normalized DNA fluorescence content inside the segmentation mask. DNA content was normalized based on M-phase cell annotations.

### EdU content quantification

We normalized EdU contents using the following procedure:

1. We first applied a median filter and thresholded the image. All pixel values less than t1 were set to t1 and all pixel values greater than t2 were set to t2 (t1 and t2 were determined on an image-by-image basis). We next normalized the image to the [0,1] range.
2. For each segmentation mask, we summed all normalized EdU values of pixels inside the mask. We then normalized the 10th and 85th percentiles of cellular EdU contents in a given gonadal arm to 0 and 1, respectively.
3. We classified cells as EdU-positive or EdU-negative by applying a manually set threshold.

Cell cycle phase indices were computed at each row by aggregating cells at that row, and computing the proportions of cells at various phases of the cell cycle (EdU-positive cells are in S-phase, EdU-negative cells with low DNA content are in G1, and EdU-negative cells with high DNA content are in G2/M).

### Morphological classification of cell phase

We cropped 2D cell segmentations to a maximum size of 34x34 pixels. We used the following features in morphological classification of cell phase:

1. Number of connected components of the thresholded foreground mask. For each segmented cell, we generated a foreground mask via Otsu thresholding of of the DNA channel. Then, we counted the number of connected components via Matlab’s regionprops command.
2. Number of pixels composing the thresholded foreground mask. For each segmented cell, we generated a foreground mask via Otsu thresholding of the DNA channel. Then, we counted the number of pixels inside the foreground mask.
3. Center-surround Haar-like features. Haar-like features are simple convolution masks that can be used to detect puncta [89]. Let r = (α,β,u,v) parameterize a rectangle such that r is composed of all points bounded by α ≤ x ≤ α + u and β ≤ y ≤ β + v. Then a Haar-like feature can be specified by two rectangles r_1_, r_2_ where r_1_ encompasses r_2_. In our application of Haar-like features, we used “center-surround” features such that r_1_, r_2_ are squares and r_2_ is positioned in the center of r_1_. In addition, we set the maximum size of r_1_ to be eight pixels wide; altogether, there are 5239 possible Haar-like features that are eight or less pixels wide in a 34x34 image frame. The image response to a Haar-like feature is the average pixel value within r_2_ minus the average pixel value within r_1_ not in r_2_.

We trained and ran SVMs using Matlab’s svmtrain and svmclassify commands. We trained SVMs using the following parameters:

1. Maximum number of iterations = 150,000
2. Tolerance = 1e-7 for the G1 classifier, 1e–8 for the S classifier, 1e–8 for the G2 classifier, 1e–8 for the M classifier.
3. Box constraint = 1e–5 for the G1 classifier, 1e–3 for the S classifier, 1e–2 for the G2 classifier, 1e–3 for the M classifier

When training and running SVMs, we split datasets randomly into equally-sized, non-intersecting training and testing subsets. In order to generate classifier statistics, we repeatedly resampled training and testing subsets.

## Availability of supporting data

Datasets comprising ~45,000 segmented worm germ cells can be downloaded from http://cinquin.org.uk/static/Parismi_datasets.tgz.

## Additional files

Additional file 1: Figure S1. Examples of varying DNA morphologies across *C. elegans* germ cells. Cells shown were taken at different phases of mitosis (A) or meiosis (B). (PDF 334 kb)
Additional file 2: Table S1. Average precision of cell detection over a variety of experimental conditions. **Table S2.** Average precision of cell detection over the whole worm gonadal arms. **Table S3.** Average overlap of cell segmentations across different experimental conditions. **Table S4.** Benchmarking EdU quantification accuracy. **Table S5.** Performance comparisons with other software. **Table S6.** Performance scored following ground truths provided by different users. **Table S7.** Number of connected components of DNA image in G1-, S-, G2-, and M-phase cells. **Table S8.** Pairwise comparisons of number of connected components in DNA images. **Table S9.** DNA spatial extent in G1-, S-, G2-, and M phase cells. **Table S10.** Pairwise comparisons of DNA spatial extent in G1-, S-, G2-, and M-phase cells. **Table S11.** Sensitivity and specificity of classifier. (PDF 114 kb)
Additional file 3: Figure S2. Cell detection via HOG features. (A) HOG weights learned from germ cells. The left two panels display the component of the weight vector learned from xy sections, while the right two panels correspond to xz sections. (B) Variation of training samples. The left panel corresponds to the 20 highest-scoring training samples and the right panel corresponds to the 20 lowest-scoring training samples. (C) Eight curated MZs are sufficient for accurate training of the cell detector. (PDF 697 kb)
Additional file 4: Figure S3. Precision-recall curves benchmarking cell center detection. (A-B) Precision-recall trade off as the detection threshold is varied for *C. elegans* MZ cells (A) and mouse embryo (B). Based on these curves, an automatic detection threshold can be chosen that yields high precision (e.g. P = 0.98); automatic detection using this threshold can be followed by manual curation using Parismi’s annotation tool to add cells missed by the detector. Red circles show recall and precision for each of the three manual segmentations performed on the same set of cells (see main text). (PDF 117 kb)
Additional file 5: Figure S4. Segmentation accuracy for *C. elegans* germ cells. Overlay of microscope image (white signal, derived from the DNA stain DAPI) with hand-constructed segmentation (green, top row) or automatic segmentation (red, bottom row). Average overlap is 74 %. 7 z slices are shown, which cover the cell visible in the center of each slice. (PDF 101 kb)
Additional file 6: Figure S5. Comparison of hand-constructed segmentation produced independently by three users. The segmentations were performed in three dimensions; the same representative slice is shown for the three users. (PDF 23 kb)
Additional file 7: Figure S6. Benchmarking of cell row counter accuracy for *C. elegans* gonadal arms. (A) Overlay of microscope image (white signal, derived from the DNA stain DAPI) with segmentations color-coded by cell row position from the distal end (left), as computed automatically using our counter. (B) Size of the MZ scored manually vs size computed through the automatic counter. A small amount of noise (0.5 cell rows) was added in order to aid visualization of overlapping data. Positions scored manually and automatically are in close agreement; average percent deviation is 9.4 %. Diagonal shown for reference in red. (PDF 152 kb)
Additional file 8: Figure S7. Example output of various programs on *C. elegans* gonadal arm. (A) Ilastik after training with ~230 brush strokes, showing original DNA signal in white, and pixels classified as foreground (respectively background) in green (respectively red). Arrow points to nuclei that do not appear separated by background pixels, and arrowhead to a set of pixels that are classified as background instead of foreground. (B-E) The same representative slice from original DNA signal (B), hand-constructed segmentation (C), Parismi segmentation (D), and MINS segmentation (E), in which segments tend to say confined to the interior of the nuclear domain delimited by DNA signal. (PDF 420 kb)
Additional file 9: Data 1. DNA morphologies of cells classified as G1-, S-, G2-, and M-phase based on DNA and EdU content. (PDF 3296 kb)
Additional file 10: Figure S8. Comparison of frozen or vibratome section segmentations. (A) Active contour segmentation of frozen sections is often inaccurate (red arrows) due to poor cell separation in the original image. (B) Vibratome sections enable clearer staining and thus more accurate segmentation (yellow arrows). (PDF 749 kb)
Additional file 11: Figure S9. Parismi GUI screenshot. (PDF 1381 kb)
Additional file 12: Figure S10. Identification of cells on the top layer of gonadal arms. (A) “Top-layeredness” θ is measured as the fraction of a cell’s pixels that project up the z axis without intersecting another cell. (B) Gonadal arm images where all (red) and top-layer only (purple) cells have been segmented. In practice, θ > 0.1 is a suitable threshold for top-layer cells. (PDF 172 kb)

## Abbreviations

AO: Average overlap
AP: Average precision
GUI: Graphical user interface
HOG: Histogram of oriented gradients
MZ: Mitotic zone
OE: Olfactory epithelium
SVM: Support vector machine

## References

[1] Chang HH, Hemberg M, Barahona M, Ingber DE, Huang S (2008). Transcriptome-wide noise controls lineage choice in mammalian progenitor cells. Nature.

[2] Zernicka-Goetz M, Huang S (2010). Stochasticity versus determinism in development: a false dichotomy?. Nat Rev Genet.

[3] Kumar RM, Cahan P, Shalek AK, Satija R, DaleyKeyser AJ, Li H, Zhang J, Pardee K, Gennert D, Trombetta JJ, Ferrante TC, Regev A, Daley GQ, Collins JJ (2014). Deconstructing transcriptional heterogeneity in pluripotent stem cells. Nature.

[4] Carpenter AE, Jones TR, Lamprecht MR, Clarke C, Kang IH, Friman O, Guertin DA, Chang JH, Lindquist RA, Moffat J, Golland P, Sabatini DM (2006). Cell Profiler: image analysis software for identifying and quantifying cell phenotypes. Genome Biol.

[5] Berthold MR, Cebron N, Dill F, Gabriel TR, Kötter T, Meinl T, Ohl P, Sieb C, Thiel K, Wiswedel B: KNIME: The Konstanz information miner. Berlin: Springer; 2008.

[6] Jones TR, Kang IH, Wheeler DB, Lindquist RA, Papallo A, Sabatini DM, Golland P, Carpenter AE (2008). Cell Profiler Analyst: data exploration and analysis software for complex image-based screens. BMC Bioinformatics.

[7] Roysam B, Shain W, Robey E, Chen Y (2008). The FARSIGHT project: associative 4D/5D image analysis methods for quantifying complex and dynamic biological microenvironments. Microscopy and Microanalysis.

[8] Peng H, Ruan Z, Long F, Simpson JH, Myers EW (2010). V3D enables real-time 3D visualization and quantitative analysis of large-scale biological image data sets. Nat Biotechnol.

[9] Sommer C, Straehle C, Kothe U, Hamprecht FA: ilastik: Interactive learning and segmentation toolkit. In 2011:230–233.

[10] Kamentsky L, Jones TR, Fraser A, Bray M-A, Logan DJ, Madden KL, Ljosa V, Rueden C, Eliceiri KW, Carpenter AE (2011). Improved structure, function and compatibility for Cell Profiler: modular high-throughput image analysis software. Bioinformatics.

[11] de Chaumont F, Dallongeville S, Chenouard N, Hervé N, Pop S, Provoost T, Meas-Yedid V, Pankajakshan P, Lecomte T, Le Montagner Y, Lagache T, Dufour A, Olivo-Marin J-C (2012). Icy: an open bioimage informatics platform for extended reproducible research. Nat Methods.

[12] Kankaanpää P, Paavolainen L, Tiitta S, Karjalainen M, Päivärinne J, Nieminen J, Marjomäki V, Heino J, White DJ (2012). BioImageXD: an open, general-purpose and high-throughput image-processing platform. Nat Methods.

[13] Schneider CA, Rasband WS, Eliceiri KW (2012). NIH Image to ImageJ: 25 years of image analysis. Nat Methods.

[14] Schindelin J, Arganda-Carreras I, Frise E, Kaynig V, Longair M, Pietzsch T, Preibisch S, Rueden C, Saalfeld S, Schmid B, Tinevez J-Y, White DJ, Hartenstein V, Eliceiri K, Tomancak P, Cardona A (2012). Fiji: an open-source platform for biological-image analysis. Nat Methods.

[15] Ollion J, Cochennec J, Loll F, Escudé C, Boudier T (2013). TANGO: a generic tool for high-throughput 3D image analysis for studying nuclear organization. Bioinformatics.

[16] Lou X, Kang M, Xenopoulos P, Muñoz-Descalzo S, Hadjantonakis A-K (2014). A Rapid and Efficient 2D/3D Nuclear Segmentation Method for Analysis of Early Mouse Embryo and Stem Cell Image Data. Stem Cell Reports.

[17] Peng H, Bria A, Zhou Z, Iannello G, Long F (2014). Extensible visualization and analysis for multidimensional images using Vaa3D. Nat Protoc.

[18] Dufour A, Liu T, Ducroz C (2015). Signal Processing Challenges in Quantitative 3-D Cell Morphology: More than meets the eye. Signal Processing Magazine, EEE.

[19] Eliceiri KW, Berthold MR, Goldberg IG, Ibáñez L, Manjunath BS, Martone ME, Murphy RF, Peng H, Plant AL, Roysam B, Stuurman N, Stuurmann N, Swedlow JR, Tomancak P, Carpenter AE (2012). Biological imaging software tools. Nat Methods.

[20] Long F, Zhou J, Peng H (2012). Visualization and analysis of 3D microscopic images. PLoS Comput Biol.

[21] Maška M, Ulman V, Svoboda D, Matula P, Matula P, Ederra C, Urbiola A, España T, Venkatesan S, Balak DMW, Karas P, Bolcková T, Streitová M, Carthel C, Coraluppi S, Harder N, Rohr K, Magnusson KEG, Jaldén J, Blau HM, Dzyubachyk O, Křížek P, Hagen GM, Pastor-Escuredo D, Jimenez-Carretero D, Ledesma-Carbayo MJ, Muñoz-Barrutia A, Meijering E, Kozubek M, Ortiz de Solorzano C (2014). A benchmark for comparison of cell tracking algorithms. Bioinformatics.

[22] Peng H, Long F, Myers EW (2009). VANO: a volume-object image annotation system. Bioinformatics.

[23] Bashar MK, Komatsu K, Fujimori T, Kobayashi TJ (2012). Automatic extraction of nuclei centroids of mouse embryonic cells from fluorescence microscopy images. PLoS ONE.

[24] Al-Kofahi Y, Lassoued W, Lee W, Roysam B (2010). Improved automatic detection and segmentation of cell nuclei in histopathology images. IEEE transactions on bio-medical engineering.

[25] Bao Z, Murray JI, Boyle T, Ooi SL, Sandel MJ, Waterston RH (2006). Automated cell lineage tracing in Caenorhabditis elegans. Proc Natl Acad Sci USA.

[26] Santella A, Du Z, Nowotschin S, Hadjantonakis A-K, Bao Z (2010). A hybrid blob-slice model for accurate and efficient detection of fluorescence labeled nuclei in 3D. BMC Bioinformatics.

[27] Chapelle O, Schölkopf B, Zien A (editors). Semi-supervised learning. Cambridge: MIT Press; 2006.

[28] Settles B: Active learning literature survey. Computer Sciences Technical Report 1648, University of Wisconsin-Madison 2009, 15.

[29] Ortiz de Solorzano C, Malladi R, Lelièvre SA, Lockett SJ (2001). Segmentation of nuclei and cells using membrane related protein markers. J Microsc.

[30] Dufour A, Shinin V, Tajbakhsh S, Guillén-Aghion N, Olivo-Marin J-C, Zimmer C (2005). Segmenting and tracking fluorescent cells in dynamic 3-D microscopy with coupled active surfaces. IEEE Trans Image Process.

[31] Dzyubachyk O, van Cappellen WA, Essers J, Niessen WJ, Meijering E (2010). Advanced level-set-based cell tracking in time-lapse fluorescence microscopy. IEEE Trans Med Imaging.

[32] Zanella C, Campana M, Rizzi B, Melani C, Sanguinetti G, Bourgine P, Mikula K, Peyriéras N, Sarti A (2010). Cells segmentation from 3-D confocal images of early zebrafish embryogenesis. IEEE Trans Image Process.

[33] Dufour A, Thibeaux R, Labruyère E, Guillén N, Olivo-Marin J-C (2011). 3-D active meshes: fast discrete deformable models for cell tracking in 3-D time-lapse microscopy. IEEE Trans Image Process.

[34] Mosaliganti KR, Noche RR, Xiong F, Swinburne IA, Megason SG (2012). ACME: automated cell morphology extractor for comprehensive reconstruction of cell membranes. PLoS Comput Biol.

[35] Delgado-Gonzalo R, Chenouard N, Unser M (2013). Spline-Based Deforming Ellipsoids for Interactive 3D Bioimage Segmentation. IEEE Trans Image Process.

[36] Lin G, Chawla MK, Olson K, Barnes CA, Guzowski JF, Bjornsson C, Shain W, Roysam B (2007). A multi-model approach to simultaneous segmentation and classification of heterogeneous populations of cell nuclei in 3D confocal microscope images. Cytometry A.

[37] Fernandez R, Das P, Mirabet V, Moscardi E, Traas J, Verdeil J-L, Malandain G, Godin C (2010). Imaging plant growth in 4D: robust tissue reconstruction and lineaging at cell resolution. Nat Methods.

[38] Olivier N, Luengo-Oroz MA, Duloquin L, Faure E, Savy T, Veilleux I, Solinas X, Débarre D, Bourgine P, Santos A, Peyriéras N, Beaurepaire E (2010). Cell lineage reconstruction of early zebrafish embryos using label-free nonlinear microscopy. Science.

[39] Long F, Peng H, Liu X, Kim SK, Myers E (2009). A 3D digital atlas of C. elegans and its application to single-cell analyses. Nat Methods.

[40] Li G, Liu T, Tarokh A, Nie J, Guo L, Mara A, Holley S, Wong STC (2007). 3D cell nuclei segmentation based on gradient flow tracking. BMC Cell Biol.

[41] Luengo-Oroz MA, Pastor-Escuredo D, Castro-Gonzalez C, Faure E, Savy T, Lombardot B, Rubio-Guivernau JL, Duloquin L, Ledesma-Carbayo MJ, Bourgine P, Peyriéras N, Santos A (2012). 3D + t morphological processing: applications to embryogenesis image analysis. IEEE Trans Image Process.

[42] Yushkevich PA, Piven J, Hazlett HC, Smith RG, Ho S, Gee JC, Gerig G (2006). User-guided 3D active contour segmentation of anatomical structures: significantly improved efficiency and reliability. Neuroimage.

[43] Hubbard EJA (2007). Caenorhabditis elegans germ line: a model for stem cell biology. Dev Dyn.

[44] Cinquin O (2009). Purpose and regulation of stem cells: a systems-biology view from the Caenorhabditis elegans germ line. J. Pathol..

[45] Chiang M, Cinquin A, Paz A, Meeds E, Price CA (2015). Welling M. Cinquin O: Control of C. elegans germline stem cell cycling speed meets requirements of design to minimize mutation accumulation. BMC Biol.

[46] Vincent L, Soille P (1991). Watersheds in digital spaces: an efficient algorithm based on immersion simulations. IEEE Trans Pattern Anal Mach Intell.

[47] Beucher S: Watershed, hierarchical segmentation and waterfall algorithm. In Mathematical morphology and its applications to image processing. Heidelberg: Springer Netherlands; 1994:69–76.

[48] Huth J, Buchholz M, Kraus JM, Schmucker M, von Wichert G, Krndija D, Seufferlein T, Gress TM, Kestler HA (2010). Significantly improved precision of cell migration analysis in time-lapse video microscopy through use of a fully automated tracking system. BMC Cell Biol.

[49] Smal I, Draegestein K, Galjart N, Niessen W, Meijering E (2008). Particle filtering for multiple object tracking in dynamic fluorescence microscopy images: application to microtubule growth analysis. IEEE Trans Med Imaging.

[50] Qu L, Long F, Liu X, Kim S, Myers E, Peng H: Simultaneous Recognition and Segmentation of Cells: Application in C. elegans. Bioinformatics 2011;27:2895-2902. 10.1093/bioinformatics/btr480 PMC3187651 21849395

[51] Maciejowski J, Ugel N, Mishra B, Isopi M, Hubbard EJA (2006). Quantitative analysis of germline mitosis in adult C. elegans. Dev Biol.

[52] Held M, Schmitz MHA, Fischer B, Walter T, Neumann B, Olma MH, Peter M, Ellenberg J, Gerlich DW (2010). Cell Cognition: time-resolved phenotype annotation in high-throughput live cell imaging. Nat Methods.

[53] Du TH, Puah WC, Wasser M (2011). Cell cycle phase classification in 3D in vivo microscopy of Drosophila embryogenesis. BMC Bioinformatics.

[54] Prewitt JM, Mendelsohn ML (1966). The analysis of cell images. Ann N Y Acad Sci.

[55] Giroud F, Gauvain C, Seigneurin D, von Hagen V (1988). Chromatin texture changes related to proliferation and maturation in erythrocytes. Cytometry.

[56] Rousselle C, Paillasson S, Robert-Nicoud M, Ronot X (1999). Chromatin texture analysis in living cells. Histochem J.

[57] Otsu N (1979). A threshold selection method from gray-level histograms. IEEE Transactions on Systems, Man and Cybernetics.

[58] Viola P, Jones M: Rapid object detection using a boosted cascade of simple features. In 2001, 1:I–511.

[59] Ersoy I, Bunyak F, Chagin V, Cardoso MC, Palaniappan K (2009). Segmentation and classification of cell cycle phases in fluorescence imaging. Med Image Comput Comput Assist Interv.

[60] Jaeger S, Palaniappan K, Casas-Delucchi CS, Cardoso MC: Classification of Cell Cycle Phases in 3D Confocal Microscopy Using PCNA and Chromocenter Features. In New York, NY, USA: ACM; 2010:412–418.

[61] Sakaue-Sawano A, Miyawaki A: Visualizing spatiotemporal dynamics of multicellular cell-cycle progressions with fucci technology. Cold Spring Harbor Protocols 2014, 2014. 10.1101/pdb.prot080408 24786503

[62] Stubbs S, Thomas N (2006). Dynamic green fluorescent protein sensors for high-content analysis of the cell cycle. Meth. Enzymol..

[63] Padfield D, Rittscher J, Roysam B (2011). Coupled minimum-cost flow cell tracking for high-throughput quantitative analysis. Med Image Anal.

[64] Padfield D, Rittscher J, Thomas N, Roysam B (2009). Spatio-temporal cell cycle phase analysis using level sets and fast marching methods. Med Image Anal.

[65] Félix M-A, Braendle C (2010). The natural history of Caenorhabditis elegans. Curr Biol.

[66] Angelo G, van Gilst MR (2009). Starvation protects germline stem cells and extends reproductive longevity in C. elegans. Science.

[67] Fukuyama M, Rougvie AE, Rothman JH (2006). C. elegans DAF-18/PTEN mediates nutrient-dependent arrest of cell cycle and growth in the germline. Curr Biol.

[68] Narbonne P, Roy R (2006). Inhibition of germline proliferation during C. elegans dauer development requires PTEN, LKB1 and AMPK signalling. Development.

[69] de Mochel NS R, Luong M, Chiang M, Javier AL, Luu E, Toshihiko F, MacGregor GR, Cinquin O, Cho KWY (2015). BMP signaling is required for cell cleavage in preimplantation-mouse embryos. Dev Biol.

[70] Chen L, Yabuuchi A, Eminli S, Takeuchi A, Lu C-W, Hochedlinger K, Daley GQ (2009). Cross-regulation of the Nanog and Cdx2 promoters. Cell Res.

[71] Dietrich J-E, Hiiragi T (2007). Stochastic patterning in the mouse pre-implantation embryo. Development.

[72] Cinquin O, Demongeot J (2005). High-dimensional switches and the modelling of cellular differentiation. J Theor Biol.

[73] Cinquin O, Page KM (2007). Generalized, switch-like competitive heterodimerization networks. Bull Math Biol.

[74] Huang S, Guo Y-P, May G, Enver T (2007). Bifurcation dynamics in lineage-commitment in bipotent progenitor cells. Dev Biol.

[75] Beites CL, Kawauchi S, Calof AL (2009). Olfactory Neuron Patterning and Specification. Dev Neurobiol.

[76] Varner VD, Nelson CM (2014). Cellular and physical mechanisms of branching morphogenesis. Development.

[77] Kawauchi S, Shou J, Santos R, Hébert JM, McConnell SK, Mason I, Calof AL (2005). Fgf8 expression defines a morphogenetic center required for olfactory neurogenesis and nasal cavity development in the mouse. Development.

[78] Boehm B, Westerberg H, Lesnicar-Pucko G, Raja S, Rautschka M, Cotterell J, Swoger J, Sharpe J (2010). The role of spatially controlled cell proliferation in limb bud morphogenesis. PLoS Biol.

[79] Pop S, Dufour AC, Le Garrec J-F, Ragni CV, Cimper C, Meilhac SM, Olivo-Marin J-C (2013). Extracting 3D cell parameters from dense tissue environments: application to the development of the mouse heart. Bioinformatics.

[80] Cinquin O, Crittenden SL, Morgan DE, Kimble J (2010). Progression from a stem cell-like state to early differentiation in the C. elegans germ line. Proc Natl Acad Sci U S A.

[81] Stallman RM, McGrath R, Smith PD: GNU Make: A program for directing recompilation, for version 3.81. Boston: Free Software Foundation; 2004.

[82] Dalal N, Triggs B: Histograms of oriented gradients for human detection. Computer Vision and Pattern Recognition, 2005. CVPR 2005. IEEE Computer Society Conference on 2005, 1:886–893 vol. 1.

[83] Felzenszwalb PF, Girshick RB, McAllester D, Ramanan D (2010). Object detection with discriminatively trained part-based models. IEEE Trans Pattern Anal Mach Intell.

[84] Steger C (1998). An unbiased detector of curvilinear structures. IEEE Trans Pattern Anal Mach Intell.

[85] Osher S, Sethian JA (1988). Fronts propagating with curvature-dependent speed: algorithms based on Hamilton-Jacobi formulations. Journal of computational physics.

[86] Adalsteinsson D, Sethian JA (1995). A fast level set method for propagating interfaces. Journal of computational physics.

[87] Kégl B, Krzyzak A, Linder T, Zeger K (2000). Learning and design of principal curves. IEEE Trans Pattern Anal Mach Intell.

[88] Dijkstra EW (1959). A note on two problems in connexion with graphs. Numerische mathematik.

[89] Jiang S, Zhou X, Kirchhausen T, Wong ST (2007). Detection of molecular particles in live cells via machine learning. Cytometry Part A.

