# Supplementary figures and images for: Analysis of in vivo single cell behavior by high throughput, human-in-the-loop segmentation of three-dimensional images
Source: BMC Bioinformatics. 2015 Nov 25;16:397. doi: 10.1186/s12859-015-0814-7 (PMC4659165; doi:10.1186/s12859-015-0814-7)

Additional Figure 1

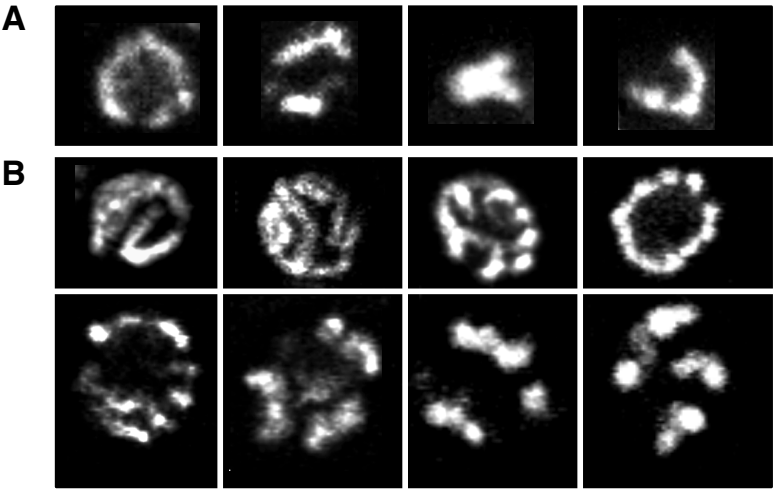

Supplement: Additional file 1: Figure S1. — Examples of varying DNA morphologies across C. elegans germ cells. Cells shown were taken at different phases of mitosis (A) or meiosis (B). (PDF 334 kb) [file 12859_2015_814_MOESM1_ESM.pdf]

Additional Figure 2

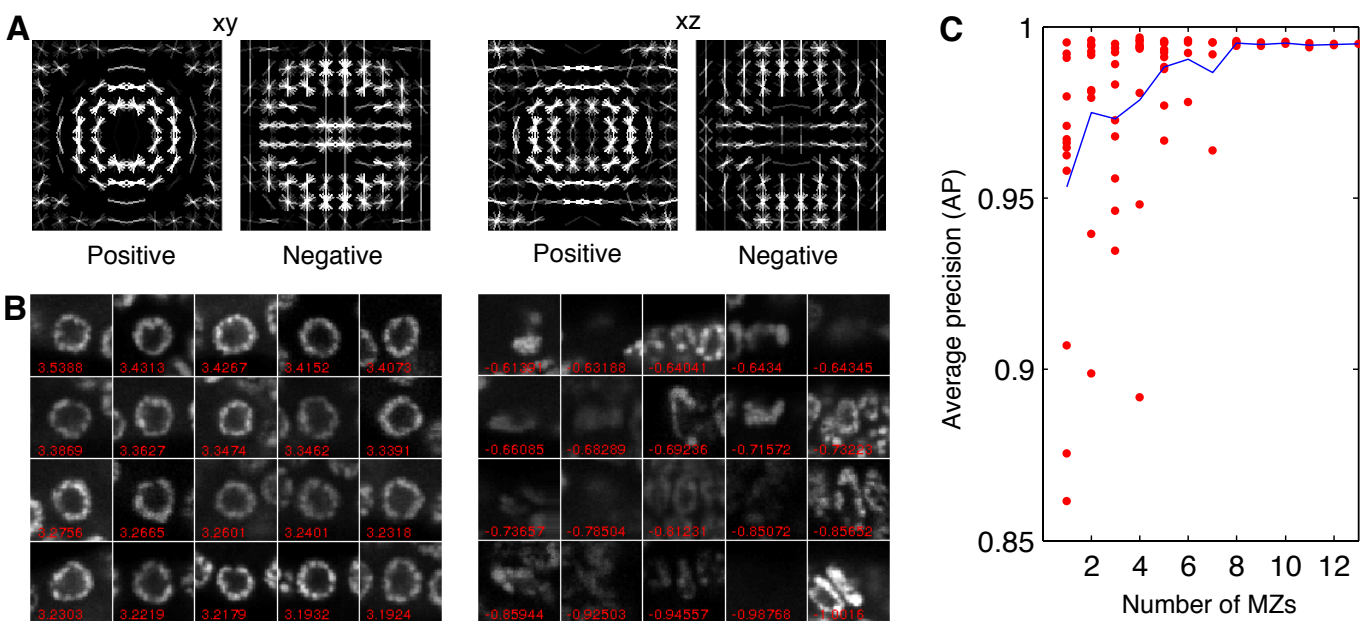

Supplement: Additional file 3: Figure S2. — Cell detection via HOG features. (A) HOG weights learned from germ cells. The left two panels display the component of the weight vector learned from xy sections, while the right two panels correspond to xz sections. (B) Variation of training samples. The left panel corresponds to the 20 highest-scoring training samples and the right panel corresponds to the 20 lowest-scoring training samples. (C) Eight curated MZs are sufficient for accurate training of the cell detector. (PDF 697 kb) [file 12859_2015_814_MOESM3_ESM.pdf]

Additional Figure 3

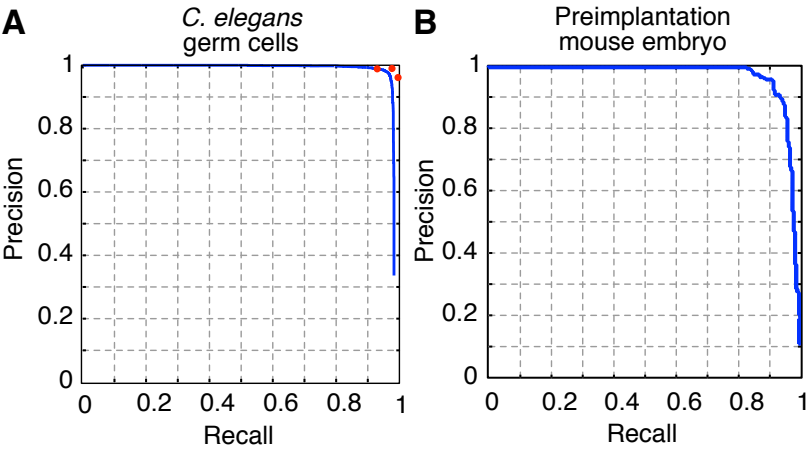

Supplement: Additional file 4: Figure S3. — Precision-recall curves benchmarking cell center detection. (A-B) Precision-recall trade off as the detection threshold is varied for C. elegans MZ cells (A) and mouse embryo (B). Based on these curves, an automatic detection threshold can be chosen that yields high precision (e.g. P = 0.98); automatic detection using this threshold can be followed by manual curation using Parismi’s annotation tool to add cells missed by the detector. Red circles show recall and precision for each of the three manual segmentations performed on the same set of cells (see main text). (PDF 117 kb) [file 12859_2015_814_MOESM4_ESM.pdf]

Additional Figure 4

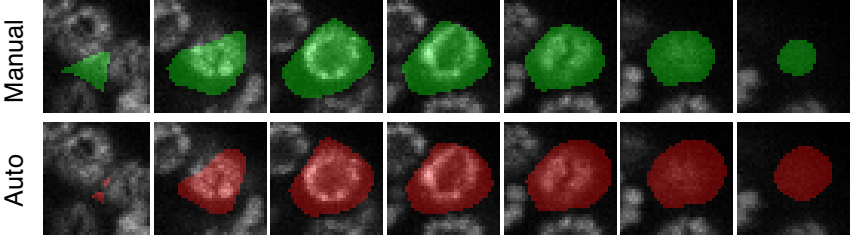

Supplement: Additional file 5: Figure S4. — Segmentation accuracy for C. elegans germ cells. Overlay of microscope image (white signal, derived from the DNA stain DAPI) with hand-constructed segmentation (green, top row) or automatic segmentation (red, bottom row). Average overlap is 74 %. 7 z slices are shown, which cover the cell visible in the center of each slice. (PDF 101 kb) [file 12859_2015_814_MOESM5_ESM.pdf]

Additional Figure 5

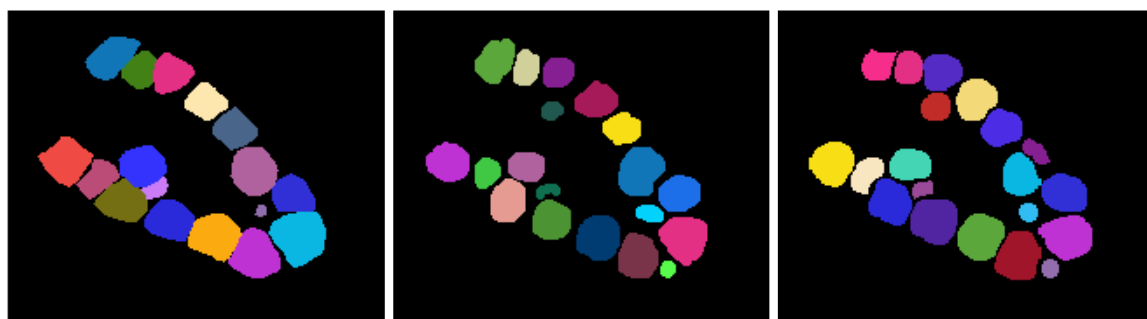

Supplement: Additional file 6: Figure S5. — Comparison of hand-constructed segmentation produced independently by three users. The segmentations were performed in three dimensions; the same representative slice is shown for the three users. (PDF 23 kb) [file 12859_2015_814_MOESM6_ESM.pdf]

Additional Figure 6

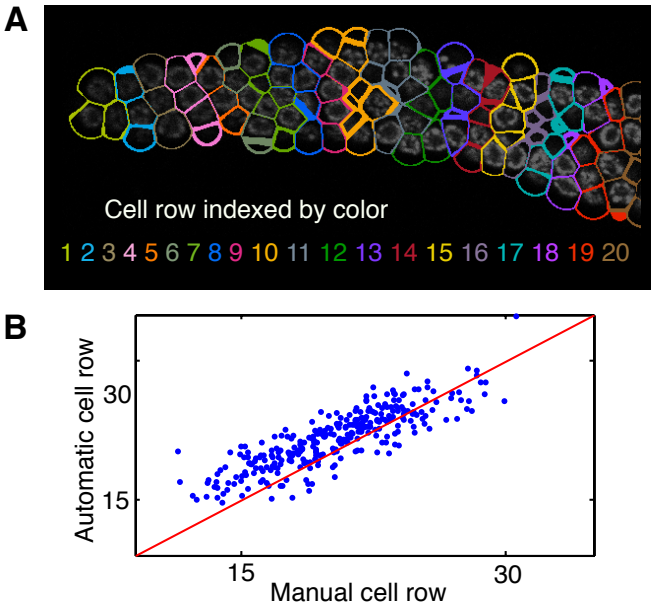

Supplement: Additional file 7: Figure S6. — Benchmarking of cell row counter accuracy for C. elegans gonadal arms. (A) Overlay of microscope image (white signal, derived from the DNA stain DAPI) with segmentations color-coded by cell row position from the distal end (left), as computed automatically using our counter. (B) Size of the MZ scored manually vs size computed through the automatic counter. A small amount of noise (0.5 cell rows) was added in order to aid visualization of overlapping data. Positions scored manually and automatically are in close agreement; average percent deviation is 9.4 %. Diagonal shown for reference in red. (PDF 152 kb) [file 12859_2015_814_MOESM7_ESM.pdf]

Additional Figure 7

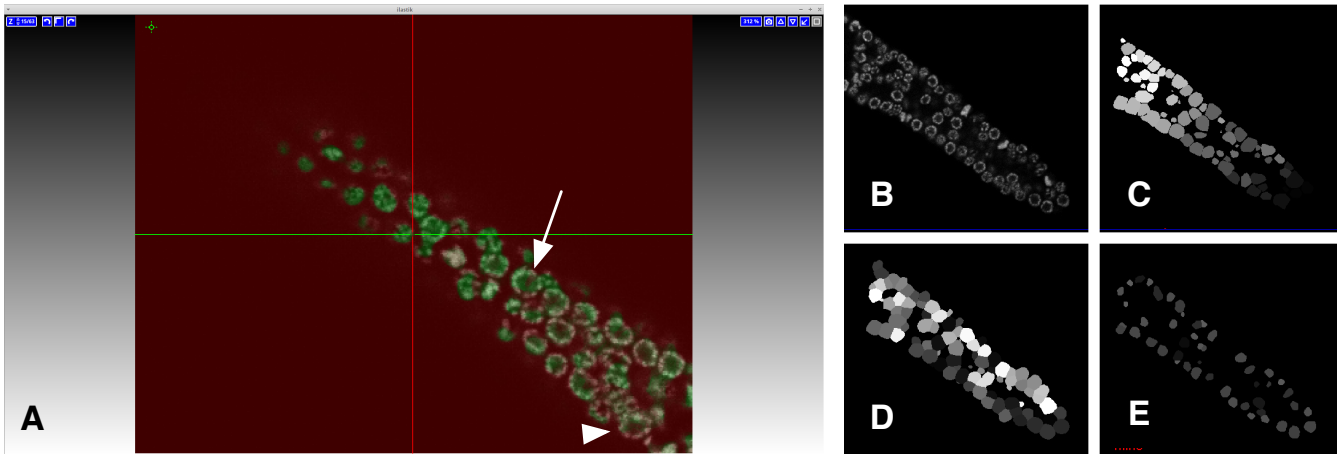

Supplement: Additional file 8: Figure S7. — Example output of various programs on C. elegans gonadal arm. (A) Ilastik after training with ~230 brush strokes, showing original DNA signal in white, and pixels classified as foreground (respectively background) in green (respectively red). Arrow points to nuclei that do not appear separated by background pixels, and arrowhead to a set of pixels that are classified as background instead of foreground. (B-E) The same representative slice from original DNA signal (B), hand-constructed segmentation (C), Parismi segmentation (D), and MINS segmentation (E), in which segments tend to say confined to the interior of the nuclear domain delimited by DNA signal. (PDF 420 kb) [file 12859_2015_814_MOESM8_ESM.pdf]

G1

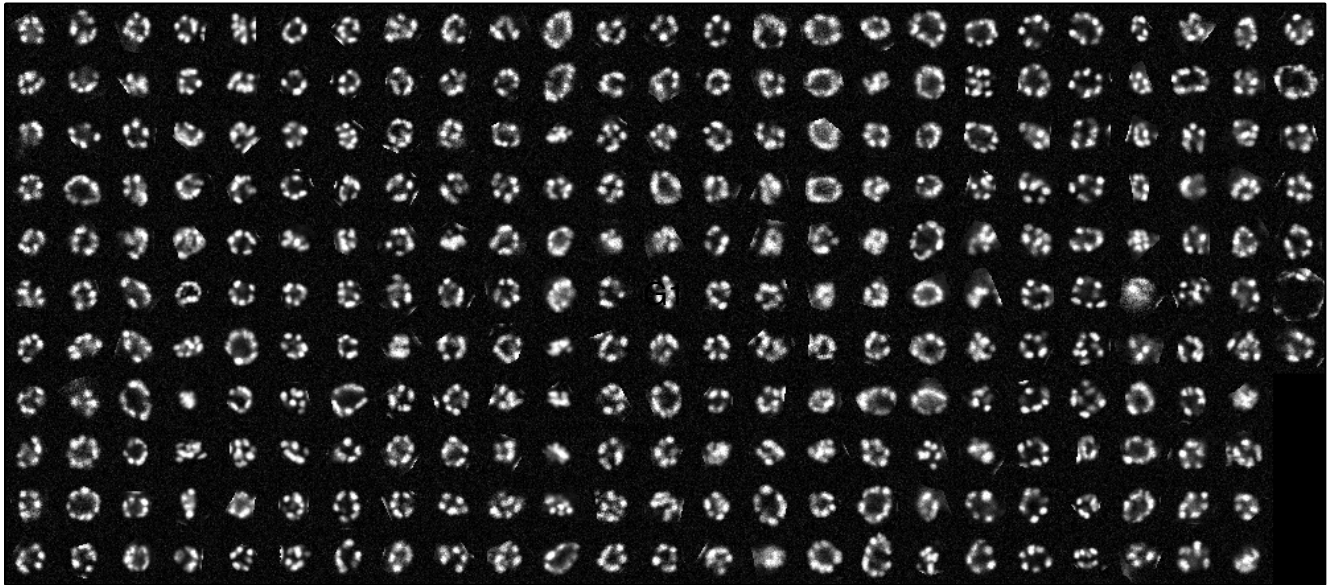

M

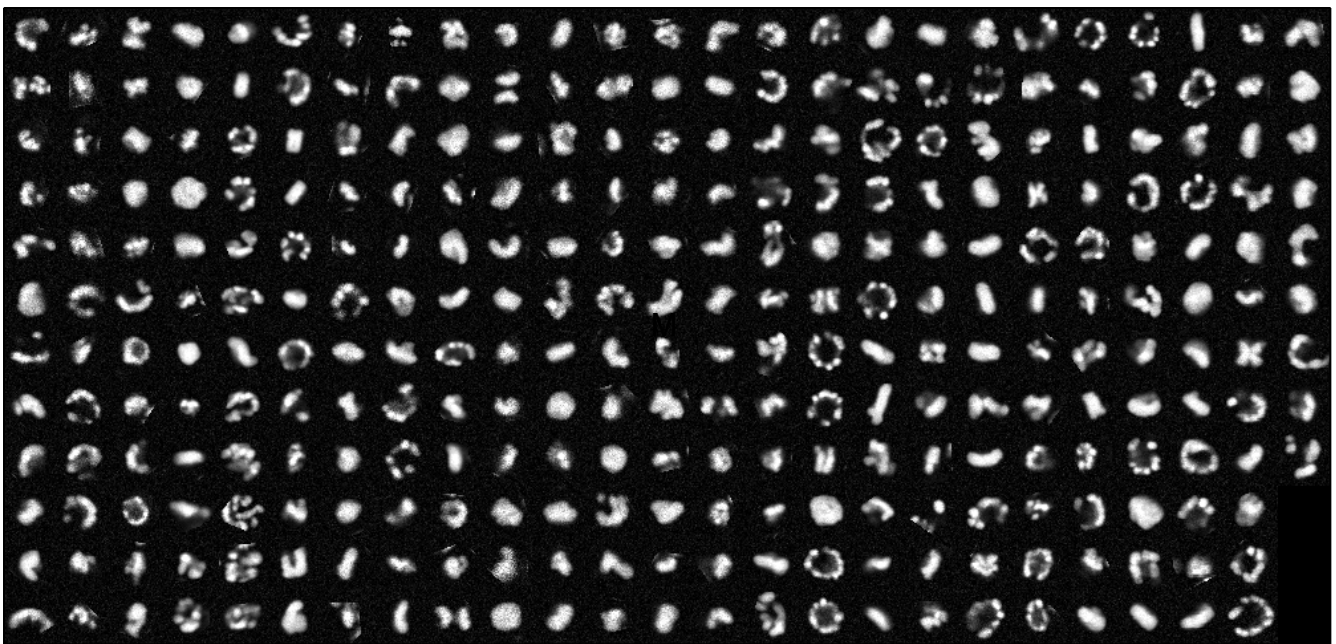

G2

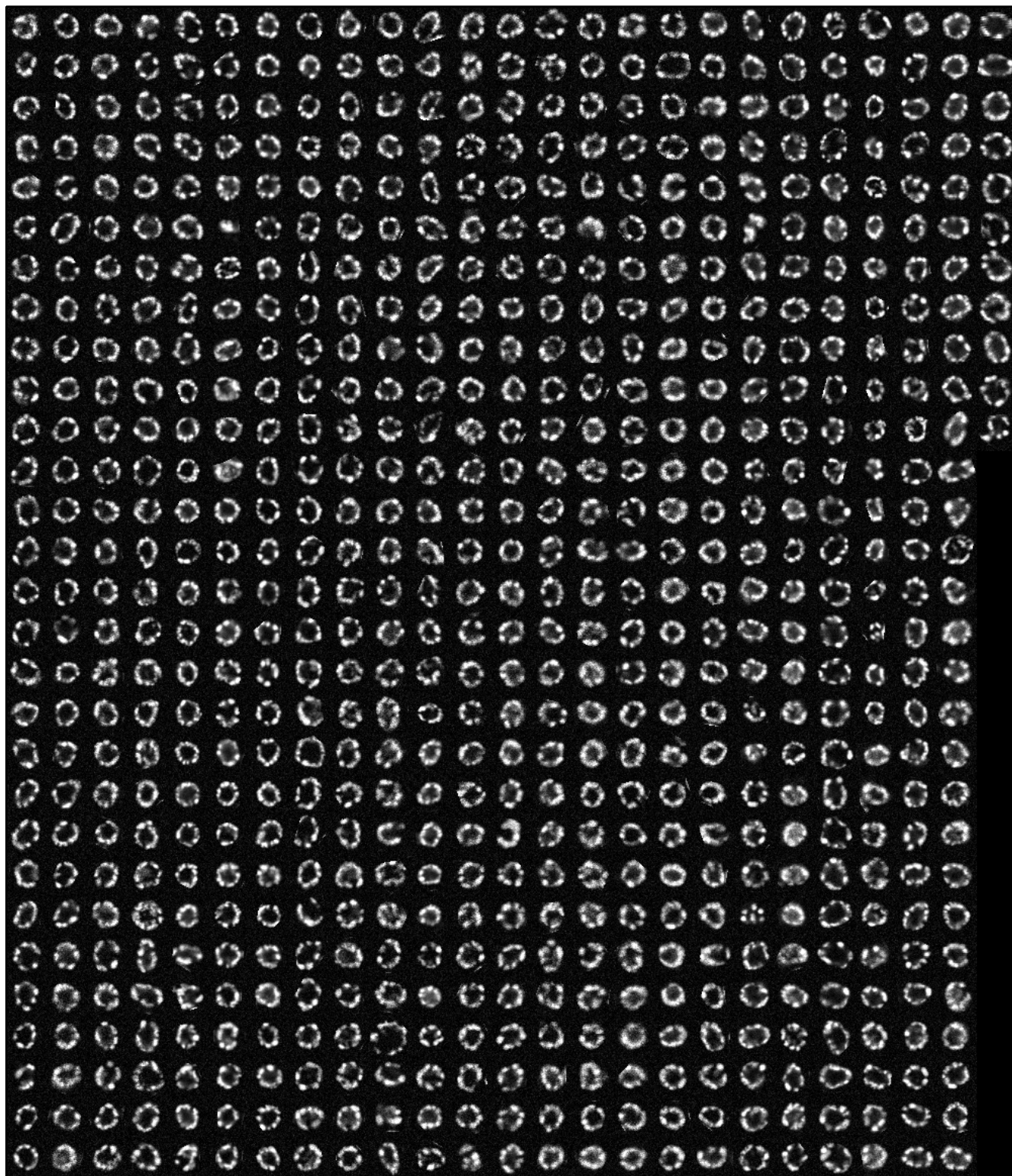

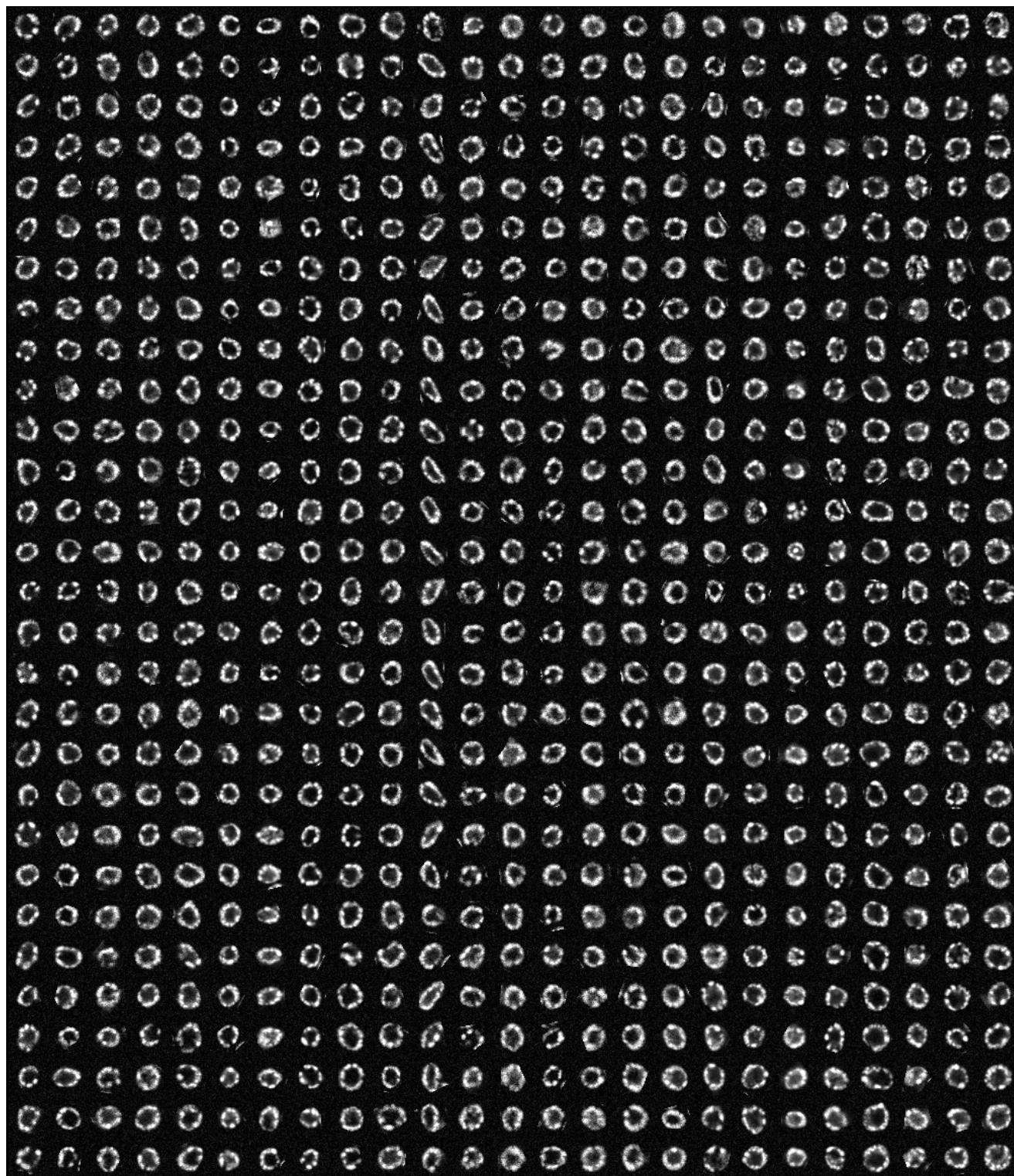

S continued

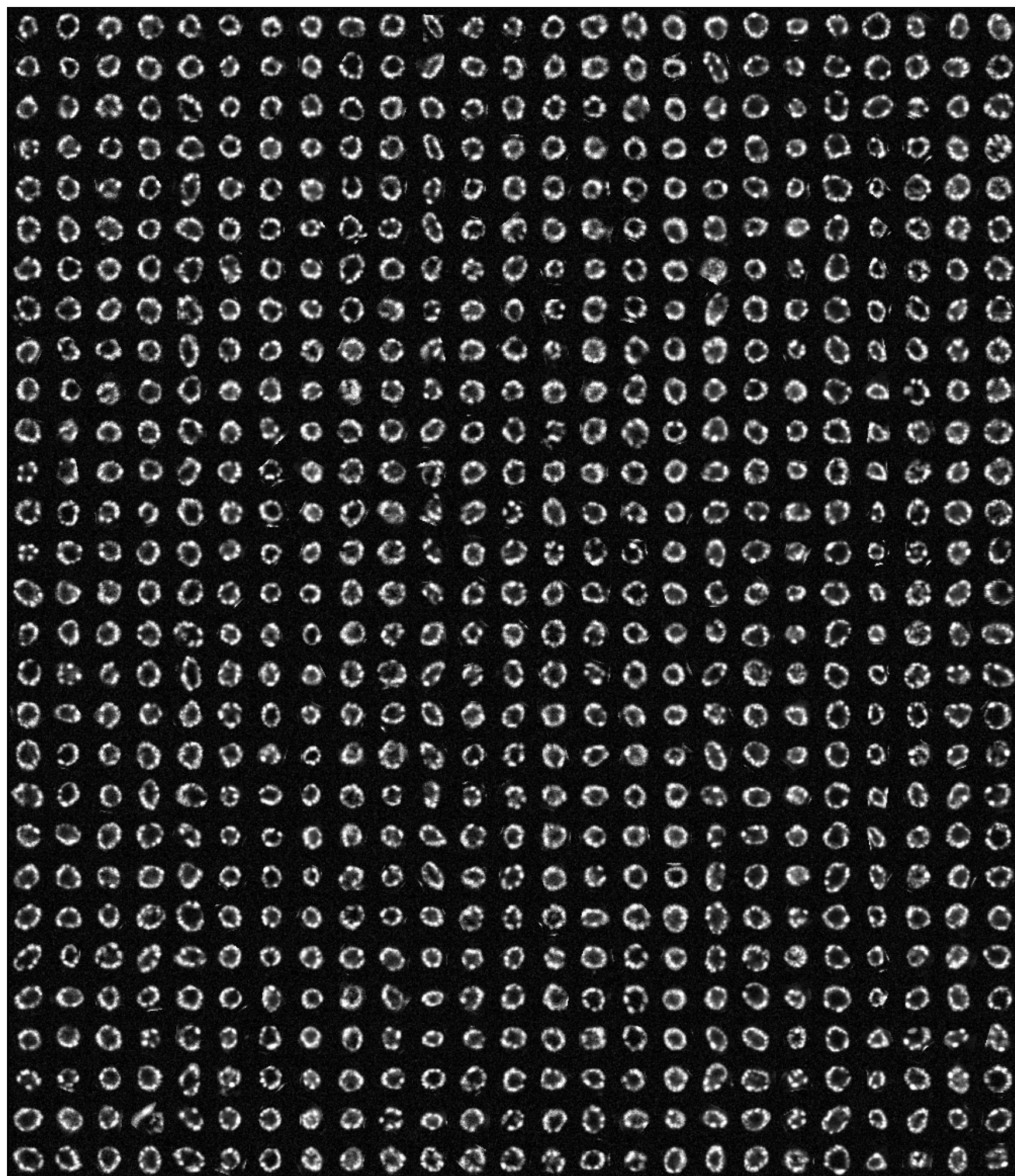

S continued

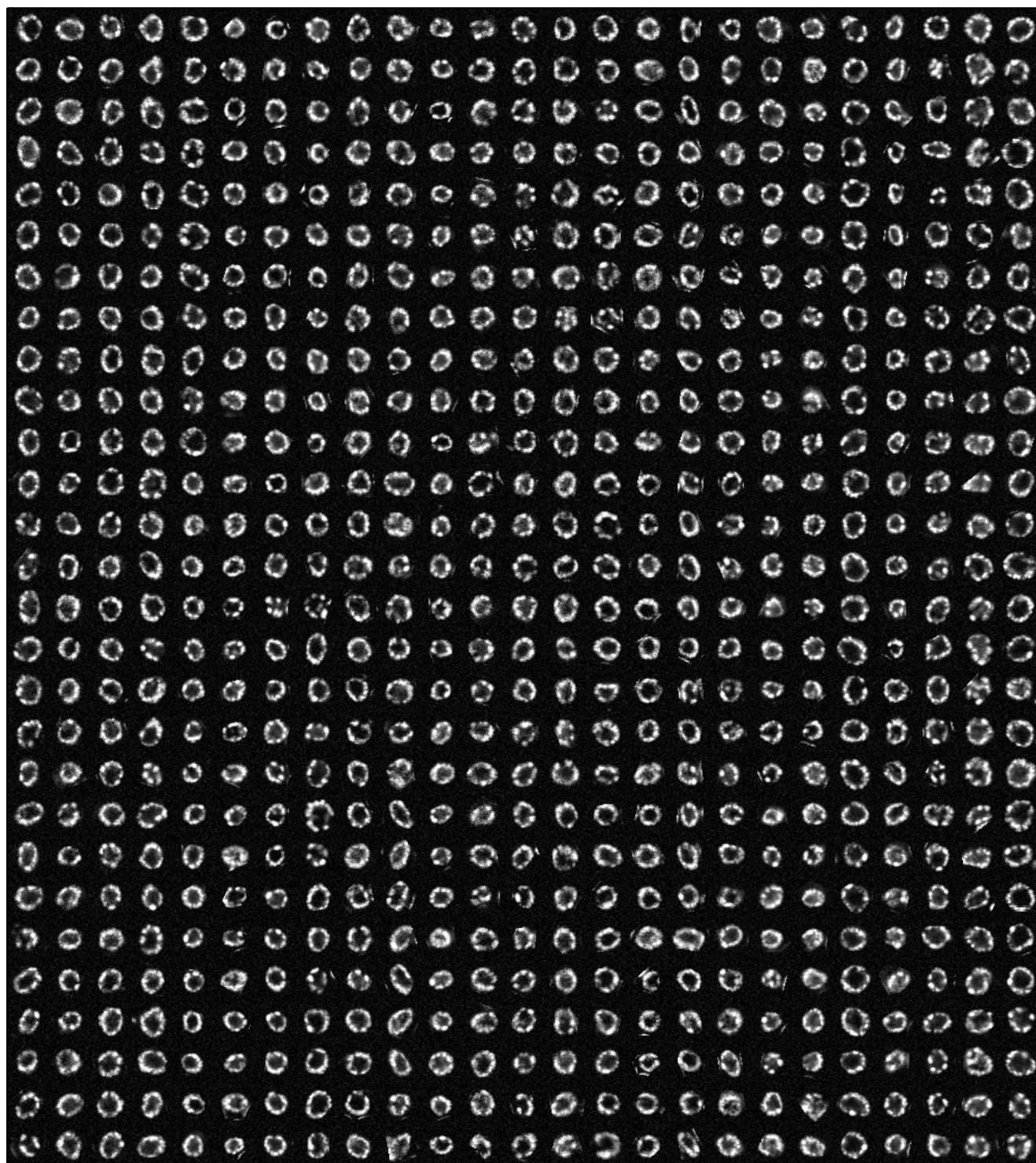

Supplement: Additional file 9: Data 1. — DNA morphologies of cells classified as G1-, S-, G2-, and M-phase based on DNA and EdU content. (PDF 3296 kb) [file 12859_2015_814_MOESM9_ESM.pdf]

Additional Figure 8

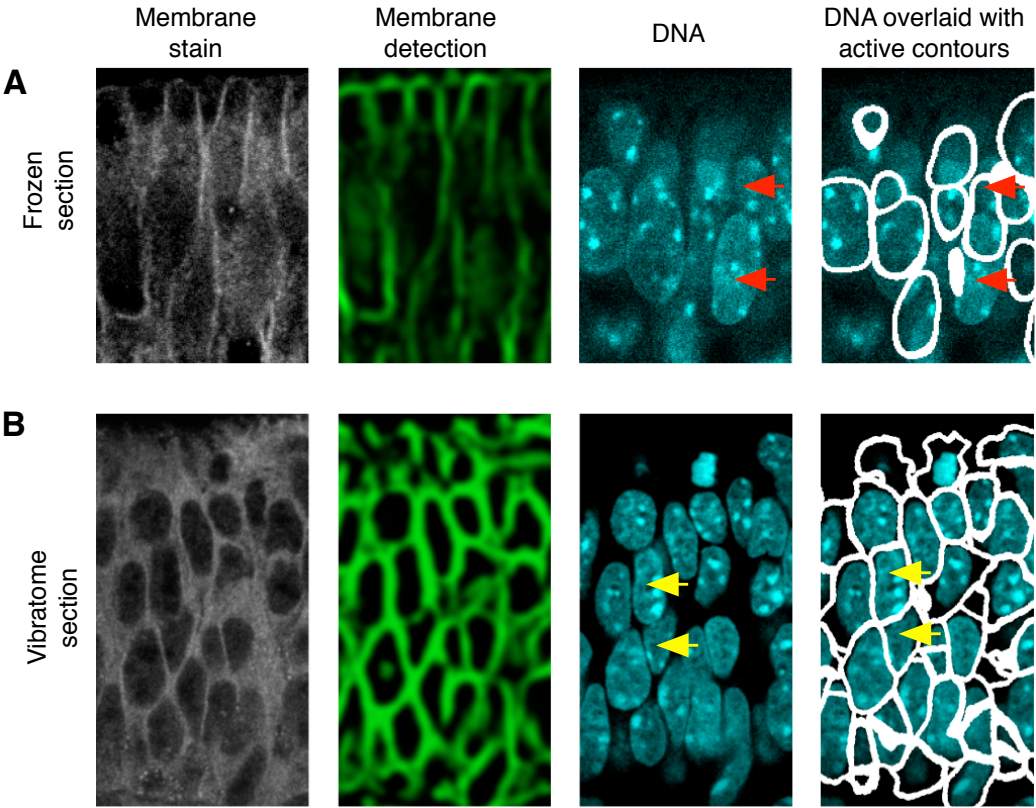

Supplement: Additional file 10: Figure S8. — Comparison of frozen or vibratome section segmentations. (A) Active contour segmentation of frozen sections is often inaccurate (red arrows) due to poor cell separation in the original image. (B) Vibratome sections enable clearer staining and thus more accurate segmentation (yellow arrows). (PDF 749 kb) [file 12859_2015_814_MOESM10_ESM.pdf]

Additional Figure 10

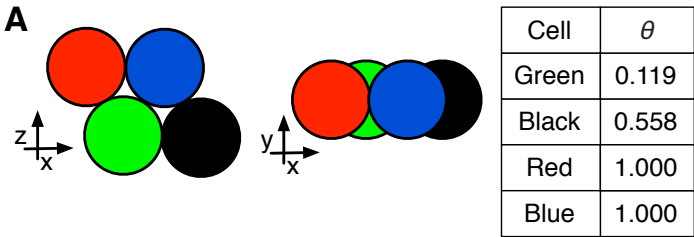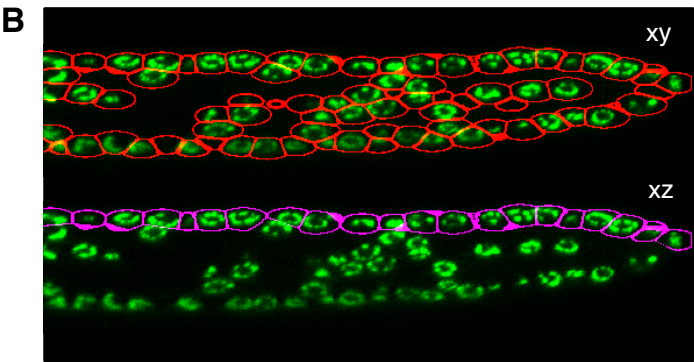

Supplement: Additional file 12: Figure S10. — Identification of cells on the top layer of gonadal arms. (A) “Top-layeredness” θ is measured as the fraction of a cell’s pixels that project up the z axis without intersecting another cell. (B) Gonadal arm images where all (red) and top-layer only (purple) cells have been segmented. In practice, θ > 0.1 is a suitable threshold for top-layer cells. (PDF 172 kb) [file 12859_2015_814_MOESM12_ESM.pdf]
