# Supplementary material for: Analysis of in vivo single cell behavior by high throughput, human-in-the-loop segmentation of three-dimensional images
Source: BMC Bioinformatics. 2015 Nov 25;16:397. doi: 10.1186/s12859-015-0814-7 (PMC4659165; doi:10.1186/s12859-015-0814-7)
Supplement: Additional file 2: Table S1. — Average precision of cell detection over a variety of experimental conditions. Table S2. Average precision of cell detection over the whole worm gonadal arms. Table S3. Average overlap of cell segmentations across different experimental conditions. Table S4. Benchmarking EdU quantification accuracy. Table S5. Performance comparisons with other software. Table S6. Performance scored following ground truths provided by different users. Table S7. Number of connected components of DNA image in G1-, S-, G2-, and M-phase cells. Table S8. Pairwise comparisons of number of connected components in DNA images. Table S9. DNA spatial extent in G1-, S-, G2-, and M phase cells. Table S10. Pairwise comparisons of DNA spatial extent in G1-, S-, G2-, and M-phase cells. Table S11. Sensitivity and specificity of classifier. (PDF 114 kb) [file 12859_2015_814_MOESM2_ESM.pdf]

Additional tables

Condition	Average precision (AP)	Number of cells
fog-2 starved 5 days	100.00%	182
N2 #1	100.00%	385
cye-1/+	99.97%	513
fog-2	99.62%	372
fog-2 mated	99.56%	276
fog-1	99.39%	335
N2 L4 + 3 days #1	98.97%	481
L4 N2	98.78%	248
N2 L4 + 3 days #2	98.75%	397
N2 #2	98.69%	10999
gld-1::gfp	96.54%	4624
inx-22; fog-2	93.91%	427

Additional Table 1. Average precision of cell detection over a variety of experimental conditions. Overall average precision across twelve replicates was 98.7% ± 2%. Unless otherwise stated, worms were not mated and were fixed at last larval stage (L4) + 1 day. N2 is wildtype.

Sample	Average precision (AP)	Number of cells
N2 gonadal arm #1	98.22%	1018
N2 gonadal arm #2	96.70%	1071
N2 gonadal arm #3	93.94%	1243
N2 gonadal arm #4	93.92%	1053
N2 gonadal arm #5	92.71%	990
N2 gonadal arm #6	88.12%	957
N2 gonadal arm #7	70.56%	1284

Additional Table 2. Average precision of cell detection over the whole worm gonadal arms. Overall average precision is 90.6% ± 9%.

Segmentation method	Guide image quality	Noise (μm)	Average overlap (AO)	AO Confidence interval (95%)
Active contour	High	0	0.62	0.61 - 0.62
Watershed	High	0	0.53	0.52 - 0.54
Voronoi	High	0	0.61	0.61 - 0.62
Active contour	Low	0	0.57	0.57 - 0.58
Watershed	Low	0	0.13	0.12 - 0.14
Voronoi	Low	0	0.61	0.61 - 0.62
Active contour	High	1	0.60	0.59 - 0.60
Watershed	High	1	0.48	0.47 - 0.50
Voronoi	High	1	0.57	0.57 - 0.58
Active contour	High	1	0.51	0.50 - 0.52
Watershed	High	1	0.33	0.31 - 0.34
Voronoi	High	1	0.45	0.44 - 0.45

Additional Table 3. Average overlap of cell segmentations across different experimental conditions. Active contours performs better than marker-controlled watershed when membrane signal is poor quality, and active contours performs better than truncated Voronoi when cell detections are not perfectly centered.

MZ sample	True positives	True negatives	False positives	False negatives	Sensitivity	Specificity	Processed cells
1	76	21	9	2	0.974	0.700	108
2	66	47	6	20	0.767	0.887	139
3	68	20	1	7	0.907	0.952	96
Aggregate	210	88	16	29	0.879	0.846	343

Additional Table 4. Benchmarking EdU quantification accuracy. Samples 1, 2, and 3 are representative of the range of EdU staining quality observed in all samples.

Software	AO	Precision	Recall
Parismi	0.60 ± 0.015	97% ± 0.73%	93% ± 5.6%
Imaris	0.37 ± 0.047	84% ± 3.5%	82% ± 3.6%
Vaa3D	0.31 ± 0.046	92% ± 1.5%	72% ± 3.7%
Ilastik	0.31 ± 0.006	89% ± 5.7%	61% ± 12%
MINS	0.30 ± 0.04	90% ± 1.4	77% ± 3.4%

Additional Table 5. Performance comparisons with other software. Performance assayed using as ground truth the same three hand-segmented *C. elegans* MZ stacks as example in Additional Figure 4 (standard deviation computed across the three stacks).

Software	User 1 ground truth	User 2 ground truth	User 3 ground truth
Parismi	AO = 0.57, P = 99%, R = 99%	AO = 0.65, P = 95%, R = 100%	AO = 0.60, P = 99%, R = 100%
Imaris	AO = 0.52, P = 96%, R = 67%	AO = 0.52, P = 96%, R = 70%	AO = 0.59, P = 96%, R = 68%
Vaa3D	AO = 0.36, P = 91%, R = 83%	AO = 0.34, P = 91%, R = 87%	AO = 0.34, P = 91%, R = 84%
Ilastik	AO = 0.36, P = 91%, R = 41%	AO = 0.39, P = 91%, R = 44%	AO = 0.35, P = 91%, R = 42%
MINS	AO = 0.40, P = 91%, R = 83%	AO = 0.34, P = 91%, R = 87%	AO = 0.37, P = 91%, R = 84%

Additional Table 6. Performance scored following ground truths provided by different users. AO: average overlap; P: precision; R: recall.

Cell cycle phase	Number of components	95% CI
G1	3.54	1.00-7.20
S	2.71	1.00-7.00
G2	3.67	1.00-8.00
M	1.90	1.00-5.00

Additional Table 7. Number of connected components of DNA image in G1-, S-, G2-, and M-phase cells. The number of connected components is calculated by thresholding DNA fluorescence content and counting the number of connected components.

Cell cycle phase	G1	S	G2	M
G1	-	9.70E-13	1.90E+00	4.40E-47
S	-	-	1.80E-33	1.60E-51
G2	-	-	-	7.60E-100
M	-	-	-	-

Additional Table 8. Pairwise comparisons of number of connected components in DNA images. Table shows pairwise rank sum p-values with Bonferroni correction.

Cell cycle phase	Spatial extent of DNA signal (μm^2)	95% CI
G1	3.31	2.14 - 4.83
S	3.92	2.61 - 5.30
G2	4.17	2.87 - 5.70
M	3.96	2.10 - 6.20

Additional Table 9. DNA spatial extent in G1-, S-, G2-, and M phase cells. The spatial extent is calculated by thresholding DNA fluorescence content and computing the area of the foreground mask.

Cell cycle phase	G1	S	G2	M
**G1**	-	3.10E-35	3.50E-48	1.10E-20
**S**	-	-	1.40E-14	3.90E+00
**G2**	-	-	-	2.40E-07
**M**	-	-	-	-

Additional Table 10. Pairwise comparisons of DNA spatial extent in G1-, S-, G2-, and M-phase cells. Table shows pairwise rank sum p-values with Bonferroni correction.

Cell cycle phase	Sensitivity	Sensitivity 95% CI	Specificity	Specificity 95% CI
**G1**	0.66	0.57 - 0.75	0.67	0.61 - 0.72
**S**	0.73	0.69 - 0.76	0.67	0.63 - 0.70
**G2**	0.76	0.70 - 0.81	0.74	0.71 - 0.76
**M**	0.71	0.68 - 0.75	0.84	0.82 - 0.87

Additional Table 11. Sensitivity and specificity of classifier. All binary classifiers have sensitivity and specificity above 66%.
