# Supplementary material for: Analysis of in vivo single cell behavior by high throughput, human-in-the-loop segmentation of three-dimensional images
Source: BMC Bioinformatics. 2015 Nov 25;16:397. doi: 10.1186/s12859-015-0814-7 (PMC4659165; doi:10.1186/s12859-015-0814-7)
Supplement: Additional file 11: Figure S9. — Parismi GUI screenshot. (PDF 1381 kb) [file 12859_2015_814_MOESM11_ESM.pdf]

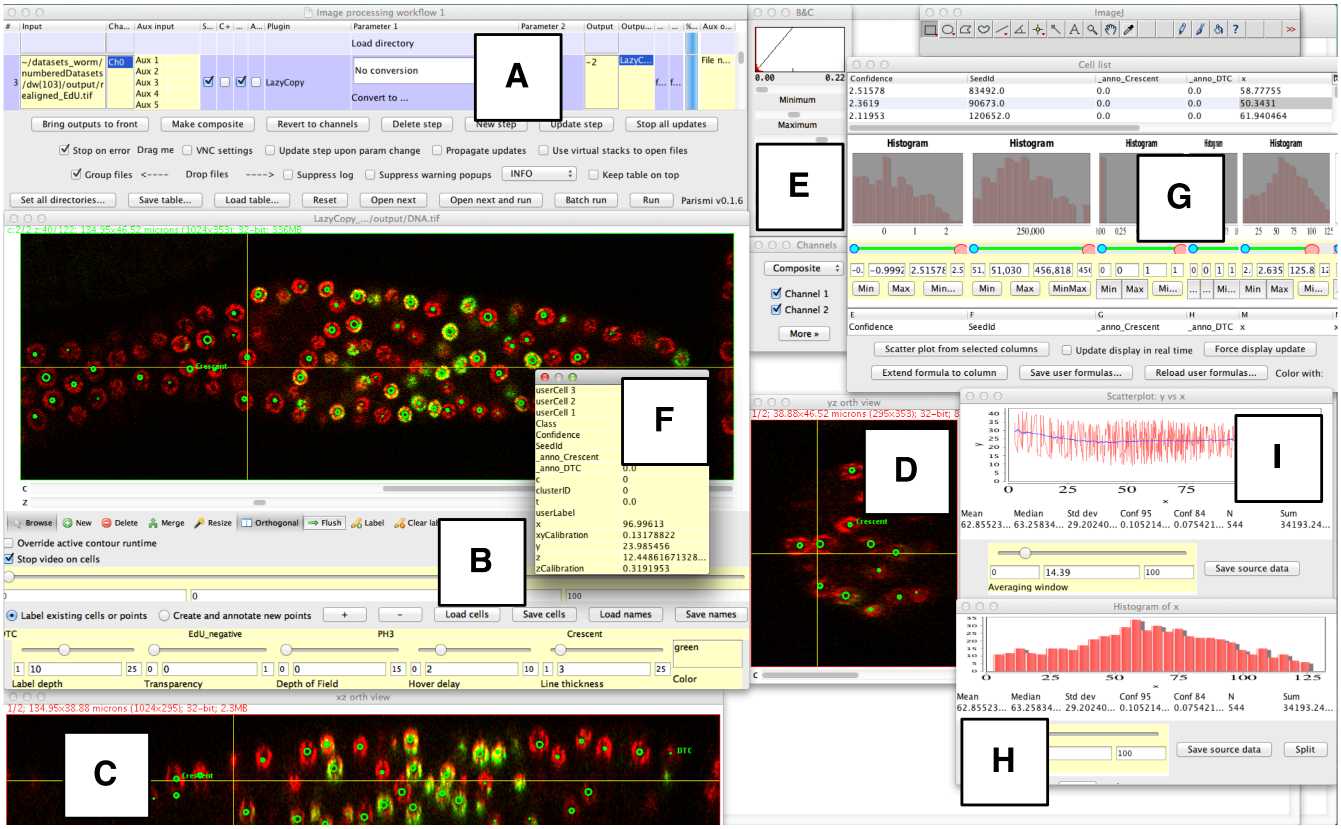

Figure S9. Parismi GUI screenshot.
(**A**) Pipeline editor. Each plugin is shown on a line of the table, and can reference outputs of other plugins with a user-specified relative or absolute offset within the table. There are currently over 100 plugins from which to chose, which are listed in a hierarchical menu (hovering on the plugin name provides a summary of plugin functionality). Type-specific parameter editors enable e.g. drag and drop of files or slider-aided interactive manipulation of numeric parameters (hovering on a parameter provides a brief overview of its purpose). “Batch run” button initiates automatic running of the pipeline across datasets, with pauses at positions specified within the pipeline to allow for user review and editing of e.g. cell detections or plugin parameters. (**B**-**D**) Cell detections (green circles) and annotations (green text) are overlaid on ImageJ image display (colors, depth of field, and various other parameters are user-adjustable), and shown both in the main xy view (B) and in orthogonal views (C-D) that are functional both for visualization and for detection and annotation editing. Parismi can control whether the original image is shown with separate channels or as a composite (here a composite is shown, with DNA signal in red and EdU signal in green). Detections can be added or removed with a single click, or moved by dragging. Annotations can be added with a single click per cell (the annotation name can be pre-selected from an editable list that is preserved across Parismi runs), or in batch by drawing a box within which all cells will receive the same annotation; all detections over a region can also be removed in the same fashion. (**E**) Standard ImageJ display controls are fully functional, and received shortcuts to minimize the amount of clicking necessary to achieve optimal display for annotation. (**F**) Hovering over annotations brings up a popup window in

which all annotation fields are listed. The window can be preserved by clicking on it; if not it disappears when the pointer moves away. (**G**-**I**) Table view allows spreadsheet-like visualization of the detections and their associated annotations, and editing of user fields. The table can be sorted on a column by clicking on the column's header. Interactive histograms summarize distributions of numerical fields. Clicking within the histogram selects a range on which detections are dynamically filtered (filtering the image overlay to only show cells that meet annotation or quantification criteria can also be achieved through the use of specific plugins). Histograms can be displayed in an independent window for closer examination (H). Pairwise plots can also be produced (I), with an optional trend-line whose averaging window can be interactively adjusted.
